# Hsa_circ_0000520 suppresses vasculogenic mimicry formation and metastasis in bladder cancer through Lin28a/PTEN/PI3K signaling

**DOI:** 10.1186/s11658-024-00627-0

**Published:** 2024-09-05

**Authors:** Chunyu Zhang, Jiao Hu, Zhi Liu, Hao Deng, Jiatong Xiao, Zhenglin Yi, Yunbo He, Zicheng Xiao, Jinliang Huang, Haisu Liang, Benyi Fan, Zhihua Wang, Jinbo Chen, Xiongbing Zu

**Affiliations:** 1 Department of Urology, Xiangya Hospital, Central South University, Changsha, China; 2 National Clinical Research Center for Geriatric Disorders, Xiangya Hospital, Central South University, Changsha, China; 3 Department of Urology, Tongji Hospital, Tongji Medical College, Huazhong University of Science and Technology, Wuhan, China; 4 Department of Urology, Hunan Provincial People’s Hospital, the First Affiliated Hospital of Hunan Normal University, Changsha, China

**Keywords:** Bladder cancer, Hsa_circ_0000520, Metastasis, Vasculogenic mimicry, PI3K/AKT pathway

## Abstract

**Background:**

Vasculogenic mimicry (VM) is a potential cause of resistance to antiangiogenic therapy and is closely related to the malignant progression of tumors. It has been shown that noncoding RNAs play an important role in the formation of VM in malignant tumors. However, the role of circRNAs in VM of bladder cancer and the regulatory mechanisms are unclear.

**Methods:**

Firstly, hsa_circ_0000520 was identified to have circular character by Sanger sequencing and Rnase R assays. Secondly, the potential clinical value of hsa_circ_0000520 was explored by quantitative real-time polymerase chain reaction (qRT-PCR) and fluorescence in situ hybridization (FISH) of clinical specimens. Thirdly, the role of hsa_circ_0000520 in bladder cancer invasion, migration, and VM formation was examined by in vivo and in vitro experiments. Finally, the regulatory mechanisms of hsa_circ_0000520 in the malignant progression of bladder cancer were elucidated by RNA binding protein immunoprecipitation (RIP), RNA pulldown, co-immunoprecipitation (co-IP), qRT-PCR, Western blot (WB), and fluorescence co-localization.

**Results:**

Hsa_circ_0000520 was characterized as a circular RNA and was lowly expressed in bladder cancer compared with the paracancer. Bladder cancer patients with high expression of hsa_circ_0000520 had better survival prognosis. Functionally, hsa_circ_0000520 inhibited bladder cancer invasion, migration, and VM formation. Mechanistically, hsa_circ_0000520 acted as a scaffold to promote binding of UBE2V1/UBC13 to Lin28a, further promoting the ubiquitous degradation of Lin28a, improving PTEN mRNA stability, and inhibiting the phosphorylation of the PI3K/AKT pathway. The formation of hsa_circ_0000520 in bladder cancer was regulated by RNA binding protein QKI.

**Conclusions:**

Hsa_circ_0000520 inhibits metastasis and VM formation in bladder cancer and is a potential target for bladder cancer diagnosis and treatment.

**Supplementary Information:**

The online version contains supplementary material available at 10.1186/s11658-024-00627-0.

## Background

Bladder cancer (BCa) is one of the more common malignant tumors in the global urological system. Epidemiological surveys estimate that, in the USA, there were approximately 82,290 new cases and 16,710 deaths in 2023, with an increasing trend in both incidence and mortality rates [1, 2]. Bladder cancer is predominantly classified into non-muscle-invasive bladder cancer (NMIBC) and muscle invasive bladder cancer (MIBC). It is characterized by high recurrence, drug resistance, and a tendency to progress and metastasize [3]. Tumor invasion and migration are closely related to angiogenesis. Some studies have shown significant efficacy of antiangiogenic targeted therapy in certain bladder cancer patients, but many still exhibit unclear efficacy or develop resistance [4–8]. The reasons for this remain unclear.

Similar to normal tissues, tumors also require an adequate blood supply to sustain their physiological activities, which is primarily accomplished through angiogenesis [9, 10]. However, in 1999, Maniotis et al. first proposed the concept of vasculogenic mimicry (VM) in melanoma, introducing a novel tumor microcirculation model that significantly differs from traditional tumor angiogenesis. VM does not rely on endothelial cells but can independently form vascular-like structures, incorporating red blood cells to provide sufficient blood supply for tumor growth [11]. VM, lacking endothelial cells, may be a key mechanism in tumor resistance to antiangiogenic therapy. Therefore, predicting and inhibiting the formation of vasculogenic mimicry in bladder cancer is particularly crucial for its treatment.

In recent years, studies have found that circular RNA (circRNA), as a type of noncoding RNA, is expressed in many cells. It possesses stable properties, is resistant to degradation by nucleases, and participates in the regulation of various physiological and pathological processes [12]. With the continuous development of technologies such as RNA sequencing, numerous differentially expressed circRNAs have been discovered in tumor tissues and cells. Their functions include acting as miRNA sponges, binding proteins, transcriptional regulation, splicing, and translation, among others [13]. In recent years, various circRNAs have been found to play significant roles in the occurrence, development, and treatment resistance of bladder cancer [14]. For example, Liu et al. identified the differentially expressed hsa_circ_0001361 in bladder cancer, which is associated with patient pathological staging and prognosis, promoting bladder cancer invasion and metastasis through the miR-491-5p/MMP9 axis [15]. Lyu et al. discovered that circUGGT2 facilitates the progression and cisplatin resistance of bladder cancer through the nonhomologous end-joining pathway [16]. In our previous research, the team screened and discovered the functional circFNTA, which can regulate its parent gene to influence KRAS activity, thereby promoting bladder cancer invasion and chemotherapy resistance [17]. These studies represent just the tip of the iceberg, and more functional circRNAs and their potential mechanisms in bladder cancer await further exploration.

In this study, we identified a circRNA derived from the *RPPH1* gene, namely hsa_circ_0000520. Hsa_circ_0000520, possessing typical circular RNA features, was downregulated in bladder cancer compared with adjacent tissues. Hsa_circ_0000520 was associated with better prognosis in bladder cancer patients. Functionally, hsa_circ_0000520 inhibited bladder cancer invasion, migration, and vasculogenic mimicry (VM) formation. Mechanistically, hsa_circ_0000520 acted as a scaffold to facilitate the binding of UBE2V1/UBC13 to Lin28a, further promoting the ubiquitination and degradation of Lin28a. This enhanced the stability of PTEN mRNA, suppressing the phosphorylation of the PI3K/AKT pathway. The formation of hsa_circ_0000520 in bladder cancer was regulated by the RNA-binding protein QKI. In summary, our study delineated the significant role of hsa_circ_0000520 in tumor progression and vasculogenic mimicry, providing a potential therapeutic target to overcome bladder cancer metastasis and resistance to antiangiogenic drugs.

## Methods

### Clinical sample acquisition

From January 2017 to August 2022, bladder cancer patients undergoing radical cystectomy or transurethral resection of bladder tumors in the Department of Urology at Xiangya Hospital, Central South University had bladder cancer tissues and adjacent noncancerous tissues collected intraoperatively. However, patients with history of other malignant tumors, severe systemic diseases, recent antitumor therapy, severe infections, or those unwilling or unable to sign informed consent forms were excluded. All specimens were promptly placed in liquid nitrogen for cryopreservation or fixed in formalin. The acquisition and utilization of tissues have obtained approval from the Ethics Committee of Xiangya Hospital, Central South University. Prior to sample collection, patients had signed relevant written informed consent forms.

### Cell culture

Human normal urothelial cells (SVHUC1) and bladder cancer cell lines (T24, UMUC3, 5637) were cultured in a cell culture incubator at 37 °C with 5% CO_2_. The cell lines were obtained from the American Type Culture Collection (ATCC). SVHUC1 was cultured in Ham’s F-12K medium (Shanghai BasalMedia Technologies Co., Ltd., Shanghai, China), T24 and 5637 were cultured in RPMI-1640 medium (Shanghai BasalMedia Technologies Co., Ltd., Shanghai, China), and UMUC3 was cultured in MEM medium containing NEAA (Shanghai BasalMedia Technologies Co., Ltd., Shanghai, China). All culture media were supplemented with 10% fetal bovine serum (ExCell Bio, Shanghai, China) and 1% penicillin–streptomycin solution (NCM Biotech, Suzhou, China).

### Cell lentiviral transfection

Lentiviruses overexpressing or knocking down hsa_circ_0000520 and Lin28a, as well as control lentiviruses, were purchased from Genechem in Shanghai, China. Initially, T24 or UMUC3 cells were seeded in six-well plates. When the cell density reached 20–30%, the supernatant was aspirated from each well. Then, 860 μl of complete culture medium, 40 μl of transfection enhancer reagent HiTransG A (Genechem, Shanghai, China), and 100 μl of virus solution at a concentration of 1 × 10^7^ TU/ml were added. The mixture was thoroughly mixed and incubated further. After 16 h of incubation at 37 °C, the culture medium containing lentivirus was aspirated, and fresh complete medium was added for continued cultivation. After 72 h of transfection, stable cell lines were established by selecting with complete medium containing 2 μg/ml puromycin for 5 days.

### Genomic DNA (gDNA) extraction

T24 or UMUC3 cells were seeded in a six-well plate, and when the cell confluence reached 90%, genomic DNA extraction was performed following the operational instructions of the SteadyPure Universal Genomic DNA Extraction Kit (Accurate Biology, Hunan, China).

### Quantitative real-time polymerase chain reaction (qRT-PCR)

For cells and tissues, RNA extraction was performed according to the instructions of the RNA extraction kit, Evo M-MLV Reverse Transcription PreMix kit, and SYBR^®^ Green Pro Taq HS PreMix qPCR kit (Accurate Biology, Hunan, China). The program was as follows: pre-denaturation at 95 °C for 30 s; PCR reaction: 95 °C for 5 s, 60 °C for 30 s. After the reaction, the amplification curve and melting curve were examined for quality assurance. The housekeeping gene β-actin was chosen as the reference, and the relative expression of the target gene was calculated using the 2^−ΔΔCt^ method.

### Western blot (WB)

Cells were seeded in a six-well plate and cultured until reaching 90% confluence. The cells were then lysed by adding a lysis buffer containing 1% protease inhibitor and phosphatase inhibitor (NCM Biotech, Suzhou, China). Moreover, 5× loading buffer (NCM Biotech, Suzhou, China) was added to the protein lysate, followed by boiling at 100 °C for 10 min. Subsequently, constant voltage electrophoresis, constant current transfer to a membrane, blocking, primary and secondary antibody incubation were performed, and the results were visualized using the Amersham ImageQuant 800 highly sensitive multifunctional imager.

### RNase R experiment

T24 or UMUC3 cells were seeded in a six-well plate and cultured until reaching 90% confluence. Total RNA was extracted, and its concentration was measured. According to the Epicentre Cat#RNR07250 kit instructions, the total RNA was divided into two portions. One portion was treated with RNase R, and the other portion, serving as a control, underwent an identical reaction system but lacking RNase R. The reaction system included 5 μg of total RNA, 2 μl of 10 × Reaction Buffer, 1 μl of RNase R, and nuclease-free water to make up the total volume to 20 μl. After thorough mixing, the reactions were carried out at 37 °C for 20 min and 80 °C for 10 min. The obtained samples were then subjected to subsequent reverse transcription and qPCR.

### Nucleocytoplasmic separation experiment

T24 or UMUC3 cells were seeded in T25 culture flasks and cultured until reaching 90% confluence. Following the instructions of the Norgen Biotek Cytoplasmic & Nuclear RNA Purification Kit, RNA samples were purified and stored at −80 °C for long-term preservation.

### Cell wound experiment

To begin with, three horizontal lines were uniformly drawn on the back of a six-well plate using a black marker. T24 or UMUC3 cells were then seeded into the plate, and upon reaching approximately 70% confluence, the complete culture medium was replaced with serum-free medium. After 12 h of incubation, a 200 μl pipette tip was used to create a vertical scratch on the cell surface, perpendicular to the lines previously drawn on the back of the plate. The scratches were observed and photographed under a microscope at 0 h and 24 h. The distance between cells was measured using ImageJ.

### Transwell experiment

#### Cell migration assay

We added 600 μl of cell culture medium containing 10% FBS to a 24-well plate. Using tweezers, the Transwell chamber was transferred to the aforementioned 24-well plate with added medium. We added 200 μl of cells diluted to an appropriate concentration to the upper chamber of the Transwell and incubated for 24 h in a cell culture incubator. After fixation with 4% paraformaldehyde, crystal violet staining, and PBS washing, we observed and captured images under a microscope.

#### Cell invasion assay

We uniformly added 50 μl of diluted Matrigel to the bottom membrane of the Transwell chamber to form a thin film. We added 600 μl of cell culture medium containing 10% FBS to a 24-well plate. Using tweezers, the Transwell chamber was transferred to the 24-well plate with added medium. We added 200 μl of cells diluted to an appropriate concentration to the upper chamber of the Transwell and incubated for 24 h in a cell culture incubator. After fixation with 4% paraformaldehyde, crystal violet staining, and PBS washing, we observed and captured images under a microscope.

### Vasculogenic mimicry formation experiment

We added 50 μl of Matrigel evenly and vertically to a 96-well plate and incubated at 37 °C for 1 h to allow gel formation. We slowly added 50 μl of diluted cell suspension to the 96-well plate with Matrigel and incubated in a 37 °C cell culture incubator for approximately 12 h, then observed and captured images under a microscope.

### Fluorescence in situ hybridization (FISH)

We designed and synthesized specific probes based on the gene information of hsa_circ_0000520 and PTEN mRNA. Slides of T24 and UMUC3 cell smears or Xiangya bladder cancer tissue cores were prepared, fixed with 4% paraformaldehyde for 20 min, and digested with proteinase K (20 μg/ml), then hybridized overnight with hsa-circ-0000520 or PTEN mRNA probes at a concentration of 6 ng/μl. We incubated with anti-digoxin-HRP antibody on slides, stained with DAPI, and observed and captured images under a fluorescence microscope.

### RNA pulldown experiment

The RNA pulldown experiment was conducted according to the Thermo Magnetic RNA–Protein Pulldown Kit instructions. We designed biotin-labeled 5′ oligonucleotide probes targeting the hsa_circ_0000520 binding site. We captured biotinylated hsa_circ_0000520 with streptavidin magnetic beads and incubated with cell lysates overnight at 4 °C. On the following day, washing and elution steps were performed. We analyzed the obtained products using mass spectrometry and validated with Western blot.

### RNA binding protein immunoprecipitation (RIP)

RIP experiments were conducted following the instructions of the Millipore Magna RIP RNA Binding Protein Immunoprecipitation Kit. We prepared T24 or UMUC3 cell lysates and incubated with 5 µg of the target protein antibody overnight at 4 °C. After treatment with proteinase K buffer, we extracted RNA and used qRT-PCR to detect RNA bound to the target protein.

### Cycloheximide (CHX) experiment

T24 or UMUC3 cells were seeded in a six-well plate until reaching 60% confluence. We added 10 μg/ml cycloheximide at 0 h, 4 h, 8 h, and 12 h to inhibit protein synthesis, extracted cell proteins, and performed Western blot to detect the expression of the target protein.

### Actinomycin d (ACTD) experiment

T24 or UMUC3 cells were seeded in a six-well plate until reaching 60% confluence. We added 5 μg/ml actinomycin d at 0 h, 4 h, 8 h, and 12 h to inhibit RNA synthesis, extracted total RNA from cells, and used qRT-PCR to detect the relative residual amount (%) of the target gene.

### Animal models

#### Bladder orthotopic tumor model

We introduced cells with luciferase using lentivirus into the desired cell line for stable expression of the luciferase gene. A bladder orthotopic tumor model was constructed using 24 female nude mice aged 6–8 weeks. We administered continuous isoflurane anesthesia and applied artificial tears to protect the eyes. We sterilized the experimental platform, fixed the anesthetized nude mice on the platform with medical tape, and disinfected. A midline incision was made in the lower abdomen to expose the bladder. A syringe was used to draw 30 μl of pretreated 50% Matrigel mixed with 50% serum-free medium containing 1 × 10^6^ T24 cells. We gently lifted the bladder wall with the syringe and slowly injected the cell suspension into the bladder wall. Successful injection was confirmed by a clear boundary between urine in the bladder and the cell suspension in the bladder wall. The abdomen was closed and the mouse was observed until it recovered from anesthesia. We injected 150 mg/kg d-luciferin into the abdominal cavity weekly and monitored tumor progression using an In Vivo Imaging System (IVIS). The mice were sacrificed after 8 weeks, the number and location of metastatic foci were recorded, and bladder orthotopic tumors were collected for further experiments.

#### Tail vein lung metastasis model

We introduced cells with luciferase using lentivirus into the desired cell line for stable expression of the luciferase gene. A tail vein lung metastasis model was established using 24 female nude mice aged 6–8 weeks. We used a mouse restrainer to immobilize the nude mice, disinfected the tail with alcohol, and used a syringe to inject 1 × 10^6^ cells/200 μl cell suspension prepared in PBS into the tail vein. We injected 150 mg/kg d-luciferin into the abdominal cavity weekly and monitored lung metastasis using an In Vivo Imaging System (IVIS). The mice were sacrificed after 8 weeks, and we recorded the number of tumors and collected lung tissues for further experiments.

### Statistical methods

GraphPad Prism 8 and SPSS 26.0 were used for statistical analysis, and experiments were repeated at least three times. Count data were analyzed using the chi-square test or Fisher’s exact probability method. Measurement data are presented as mean ± standard deviation (*x* ± *s*). Independent-sample *t*-test was used for comparing differences between two groups, and one-way analysis of variance (ANOVA) was used for comparing differences among three groups or more. Survival data were analyzed using the log-rank test and Kaplan–Meier survival analysis. A two-tailed *P*-value < 0.05 indicated a statistically significant difference.

## Results

### Hsa_circ_0000520 is downregulated in bladder cancer and correlates with better prognosis

**Fig. 1:**
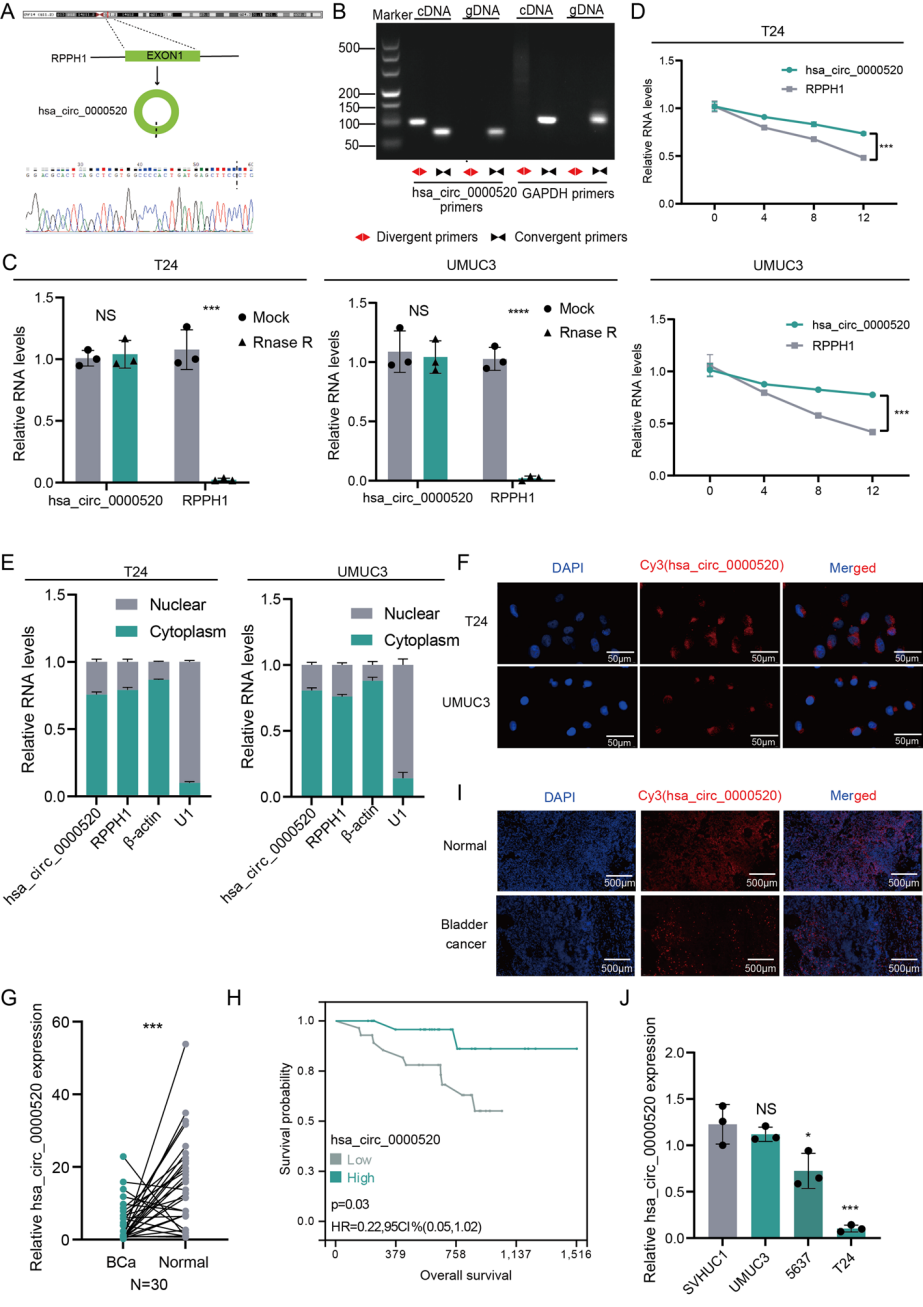
Hsa_circ_0000520 is downregulated in bladder cancer and correlates with better prognosis. **A** Confirmation of the splicing site sequence of hsa_circ_0000520 by Sanger sequencing. **B** Gel electrophoresis of PCR products obtained by amplifying hsa_circ_0000520 with reverse and forward primers. **C** qRT-PCR analysis of RNA levels of hsa_circ_0000520 and RPPH1 in T24 or UMUC3 cell lines after RNase R treatment. **D** qRT-PCR analysis of RNA abundance of hsa_circ_0000520 and RPPH1 in T24 or UMUC3 cell lines after treatment with actinomycin d for 0, 4, 8, and 12 h. **E** qRT-PCR analysis of the RNA levels of hsa_circ_0000520, β-actin, and U1 in the nucleus and cytoplasm after nuclear–cytoplasmic separation of T24 or UMUC3 cell lines. **F** Fluorescence in situ hybridization (FISH) analysis of the localization of hsa_circ_0000520 in bladder cancer cells. **G** qRT-PCR analysis of hsa_circ_0000520 RNA levels in 30 pairs of bladder cancer and adjacent tissues. **H** Kaplan–Meier survival analysis of the correlation between hsa_circ_0000520 and the overall survival of bladder cancer patients. **I** FISH analysis of differential expression of hsa_circ_0000520 in bladder cancer and adjacent tissues in tissue microarrays. **J** qRT-PCR analysis of the expression levels of hsa_circ_0000520 in normal bladder epithelial cells and bladder cancer cell lines. β-actin served as an internal reference. *P < 0.05; **P < 0.01; ***P < 0.001; ****P < 0.0001

To explore the expression profile of circular RNAs (circRNAs) in bladder cancer, we conducted a differential expression analysis of circRNA sequencing data (GSE92675) from bladder cancer tissues and their matched adjacent tissues (*n* = 3). Among the candidate differentially expressed genes, hsa_circ_0000520, derived from the exonic variable splicing of the *RPPH1* gene with a length of 123 nt, drew our attention. The circular structure of hsa_circ_0000520 was confirmed by reverse transcription polymerase chain reaction (RT-PCR) using primers designed for both reverse and forward directions. Sanger sequencing validated its consistency with the circBase database annotation (http://www.circbase.org/) (Fig. 1A). RT-PCR with reverse or forward primers confirmed the circular structure of hsa_circ_0000520, showing amplification in cDNA but not in genomic DNA (gDNA) (Fig. 1B). Ribonuclease R (RNase R) is a ribonuclease enzyme that selectively degrades linear RNAs while sparing circular RNAs. Treatment with RNase R in T24 or UMUC3 cells demonstrated the resistance of hsa_circ_0000520 to RNase R digestion (Fig. 1C). Stability analysis of hsa_circ_0000520 and its linear form in T24 or UMUC3 cells treated with actinomycin d revealed that hsa_circ_0000520 is more stable than RPPH1 (Fig. 1D). Additionally, nuclear–cytoplasmic separation experiments and fluorescence in situ hybridization (FISH) results indicated that hsa_circ_0000520 predominantly localizes in the cytoplasm of T24 and UMUC3 cells (Fig. 1E, F). These results suggest that hsa_circ_0000520 is stably expressed in bladder cancer cells.

To further validate the abnormal expression of hsa_circ_0000520 in bladder cancer, qRT-PCR was employed to detect the expression of hsa_circ_0000520 in 30 pairs of bladder cancer and adjacent tissue samples. The results showed significant downregulation of hsa_circ_0000520 in bladder cancer tissues (Fig. 1G). FISH experiments on bladder cancer tissue microarrays also demonstrated consistent results (Fig. 1I). In addition, qRT-PCR was used to assess an additional 56 bladder cancer specimens, which were divided into high and low expression groups based on the median expression level of hsa_circ_0000520, to analyze the relationship between hsa_circ_0000520 and clinical pathological features as well as prognosis of bladder cancer patients (Supplementary Table 2). The results indicated that hsa_circ_0000520 was significantly associated with lower tumor grades, with a *P*-value of < 0.001. However, there was no significant difference in hsa_circ_0000520 expression levels with respect to age, gender, stage, T stage, N stage, and M stage, which may be attributed to the limited sample size, especially with only two cases in the M stage 1 subgroup, leading to unreliable results. Furthermore, compared with patients with higher hsa_circ_0000520 expression levels, patients with lower hsa_circ_0000520 expression levels exhibited poorer overall survival (OS) (Fig. 1H). When compared with normal human uroepithelial cells (SVHUC1), hsa_circ_0000520 was downregulated in bladder cancer cell lines T24, UMUC3, and 5637 (Fig. 1J). In summary, these results suggest that hsa_circ_0000520 is downregulated in bladder cancer cells and tissues, significantly correlates with bladder cancer grading, and may serve as an independent prognostic factor for predicting clinical outcomes in bladder cancer patients.

### Clinical specimen analysis reveals a significant negative correlation between hsa_circ_0000520 and vasculogenic mimicry (VM) formation

The formation of VM was further evaluated in bladder cancer tissue microarrays, which included 47 bladder cancer tissues along with paired adjacent tissues for 33 of these bladder cancer tissues. VM formation was identified based on CD31 negativity and periodic acid–Schiff (PAS) positivity. Representative images of typical VM morphology and endothelial vessels are shown in Supplementary Fig. 1A. A strong correlation between VM formation and hsa_circ_0000520 expression was observed (Supplementary Fig. 1B, C). Bladder cancer samples with high hsa_circ_0000520 expression exhibited fewer VM formations, while those with low hsa_circ_0000520 expression showed more VM formations. These clinical data suggest a significant negative correlation between hsa_circ_0000520 and VM formation in bladder cancer.

### In vitro experiments reveal that hsa_circ_0000520 inhibits bladder cancer cell invasion, migration, and vasculogenic mimicry (VM) formation

**Fig. 2:**
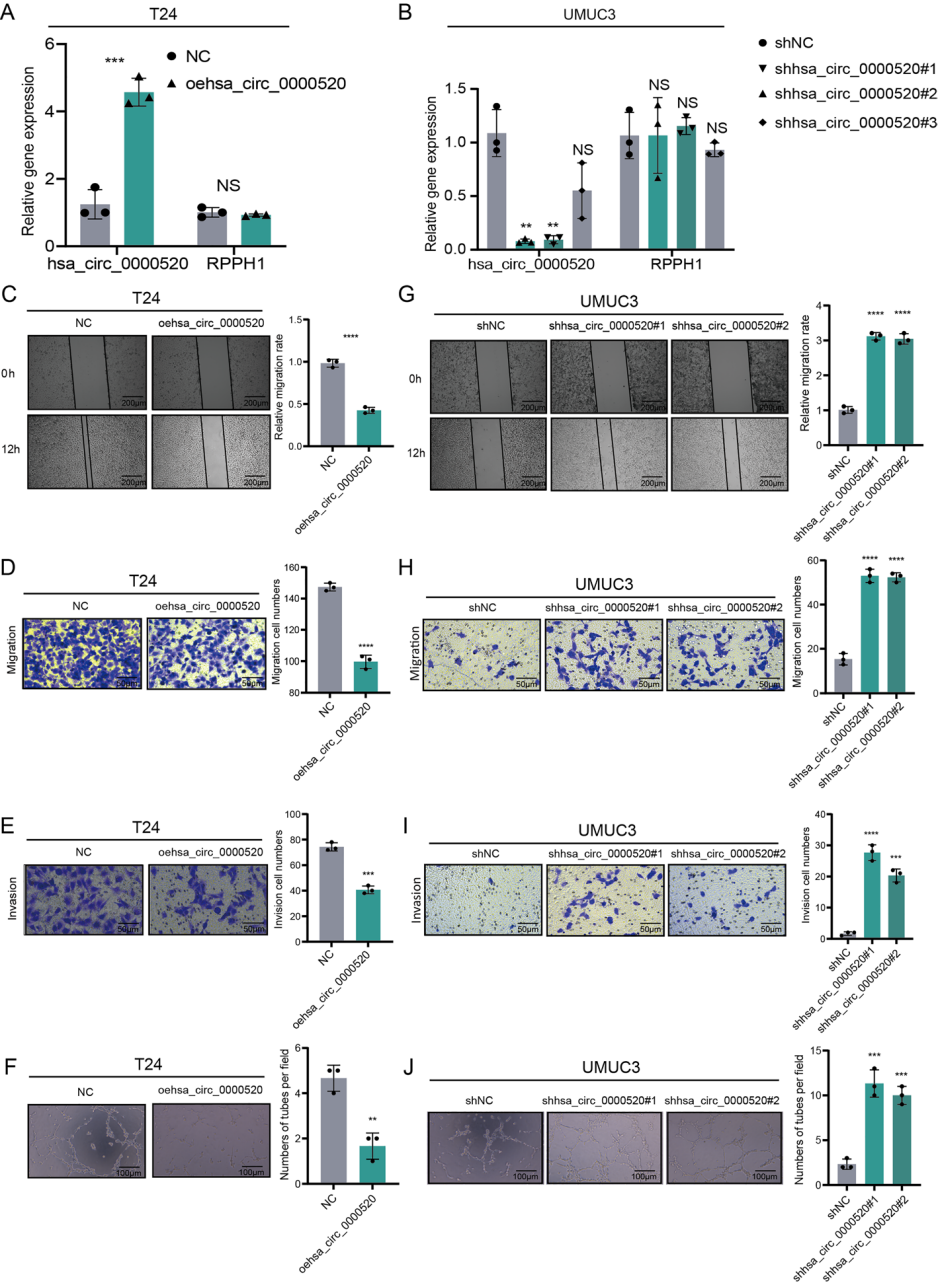
In vitro experiments reveal that hsa_circ_0000520 inhibits bladder cancer cell invasion, migration, and VM formation. **A** qRT-PCR analysis of the transfection efficiency of overexpressed hsa_circ_0000520 lentivirus in T24 cells. **B** qRT-PCR analysis of the transfection efficiency of knocked down hsa_circ_0000520 lentivirus in UMUC3 cells. **C** Scratch assay analyzing the impact of hsa_circ_0000520 overexpression on the migration ability of T24 cells. **D** Transwell assay analyzing the effect of hsa_circ_0000520 overexpression on the migration ability of T24 cells. **E** Transwell assay analyzing the effect of hsa_circ_0000520 overexpression on the invasion ability of T24 cells. **F** VM formation assay analyzing the impact of hsa_circ_0000520 overexpression on the VM formation ability of T24 cells. **G** Scratch assay analyzing the effect of knocked down hsa_circ_0000520 on the migration ability of UMUC3 cells. **H** Transwell assay analyzing the effect of knocked down hsa_circ_0000520 on the migration ability of UMUC3 cells. **I** Transwell assay analyzing the effect of knocked down hsa_circ_0000520 on the invasion ability of UMUC3 cells. **J** VM formation assay analyzing the effect of knocked down hsa_circ_0000520 on the VM formation ability of UMUC3 cells. β-actin serves as an internal reference. *P < 0.05; **P < 0.01; ***P < 0.001; ****P < 0.0001

To investigate the role of hsa_circ_0000520 in the progression of bladder cancer, we separately transduced T24 and UMUC3 cells with overexpression or knockdown viruses of hsa_circ_0000520. qRT-PCR experiments were conducted to determine the efficiency of overexpression and knockdown (Fig. 2A, B). The results showed that the expression of hsa_circ_0000520 was affected without altering the expression of RPPH1, confirming the specificity of the hsa_circ_0000520 lentivirus. Subsequently, we selected sh1-hsa_circ_0000520 and sh2-hsa_circ_0000520 with more pronounced knockdown efficiency for subsequent loss-of-function experiments. Scratch and Transwell assays indicated that overexpression of hsa_circ_0000520 significantly reduced the number of invasive and migratory bladder cancer cells (Fig. 2C, D). Vasculogenic mimicry formation experiments demonstrated that overexpression of hsa_circ_0000520 significantly inhibited VM formation in bladder cancer cells (Fig. 2F). Loss-of-function experiment results indicated that downregulation of hsa_circ_0000520 significantly promoted the aforementioned phenotypes (Fig. 2G–J). These data suggest that hsa_circ_0000520 acts as a tumor suppressor in bladder cancer, inhibiting invasion, migration, and VM formation in vitro.

### Hsa_circ_0000520 binds to Lin28a and reduces its protein stability

**Fig. 3:**
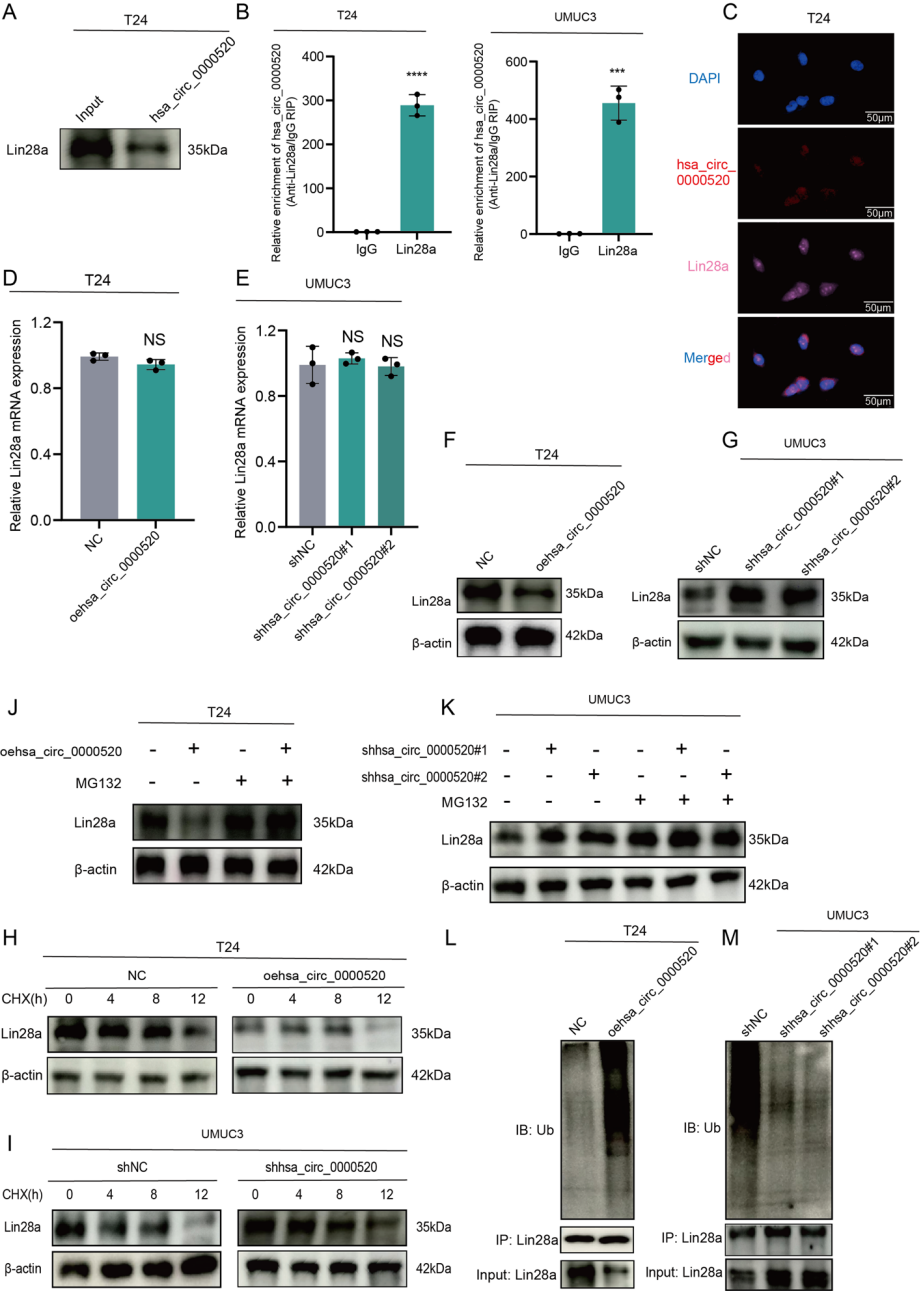
Hsa_circ_0000520 binds to Lin28a and reduces its protein stability. **A** RNA pulldown product validation by WB confirms the pulldown of Lin28a by hsa_circ_0000520. **B** RIP reverse analysis demonstrates the binding between Lin28a and hsa_circ_0000520. **C** Fluorescence colocalization analysis reveals the co-localization of hsa_circ_0000520 with Lin28a. **D**, **E** qRT-PCR analysis of the impact of overexpression and knockdown of hsa_circ_0000520 on Lin28a mRNA levels. **F**, **G** WB analysis of the effect of overexpression and knockdown of hsa_circ_0000520 on Lin28a protein levels. **H** WB analysis of changes in Lin28a protein levels after streptavidin treatment in the overexpression group of hsa_circ_0000520. **I** WB analysis of changes in Lin28a protein levels after streptavidin treatment in the knockdown group of hsa_circ_0000520. **J** WB analysis of changes in Lin28a protein levels after MG132 treatment in the overexpression group of hsa_circ_0000520. **K** WB analysis of changes in Lin28a protein levels after MG132 treatment in the knockdown group of hsa_circ_0000520. **L** Co-IP analysis of changes in Lin28a ubiquitination levels after MG132 treatment in the overexpression group of hsa_circ_0000520. **M** Co-IP analysis of changes in Lin28a ubiquitination levels after MG132 treatment in the knockdown group of hsa_circ_0000520. β-actin serves as an internal reference. *P < 0.05; **P < 0.01; ***P < 0.001; ****P < 0.0001

To elucidate the molecular mechanism by which hsa_circ_0000520 functions in bladder cancer cells, RNA pulldown experiments with biotin-labeled hsa_circ_0000520 probes were conducted to explore potential proteins interacting with hsa_circ_0000520. Initially, RNA pulldown experiments were performed using biotin-labeled hsa_circ_0000520 probes in T24 cells. Subsequent mass spectrometry analysis revealed Lin28a as a potential protein interacting with hsa_circ_0000520 in bladder cancer cells (Supplementary Table 3). Further validation of this interaction was conducted using Western blotting (WB), demonstrating the presence of Lin28a in the hsa_circ_0000520 pulldown protein complex (Fig. 3A). This interaction was further confirmed through RNA immunoprecipitation (RIP) experiments (Fig. 3B). Additionally, fluorescence co-localization analysis revealed that hsa_circ_0000520 and Lin28a co-localized in the cytoplasm of T24 cells (Fig. 3C). While the mRNA levels of Lin28a did not significantly change owing to hsa_circ_0000520, overexpression of hsa_circ_0000520 led to a significant reduction in Lin28a protein levels, whereas knockdown of hsa_circ_0000520 increased Lin28a protein levels (Fig. 3D–G). These results suggest that hsa_circ_0000520 may interact with Lin28a protein, leading to its destabilization. Cycloheximide (CHX) experiments showed a time-dependent decrease in Lin28a protein levels upon CHX treatment, and overexpression of hsa_circ_0000520 facilitated this process, resulting in a shortened half-life of Lin28a protein (Fig. 3H, I). Moreover, we found that overexpression of hsa_circ_0000520 significantly reduced Lin28a protein levels, and this reduction could be rescued by the specific proteasome inhibitor MG132 (Fig. 3J, K). Following treatment with MG132, the ubiquitination levels of Lin28a were examined, revealing that hsa_circ_0000520 significantly increased the ubiquitination levels of Lin28a (Fig. 3L, M). These results suggest that hsa_circ_0000520 enhances ubiquitin/proteasome-dependent degradation, thereby reducing the stability of Lin28a.

### Hsa_circ_0000520 serves as a scaffold to facilitate UBE2V1/UBC13-mediated degradation of Lin28a

**Fig. 4:**
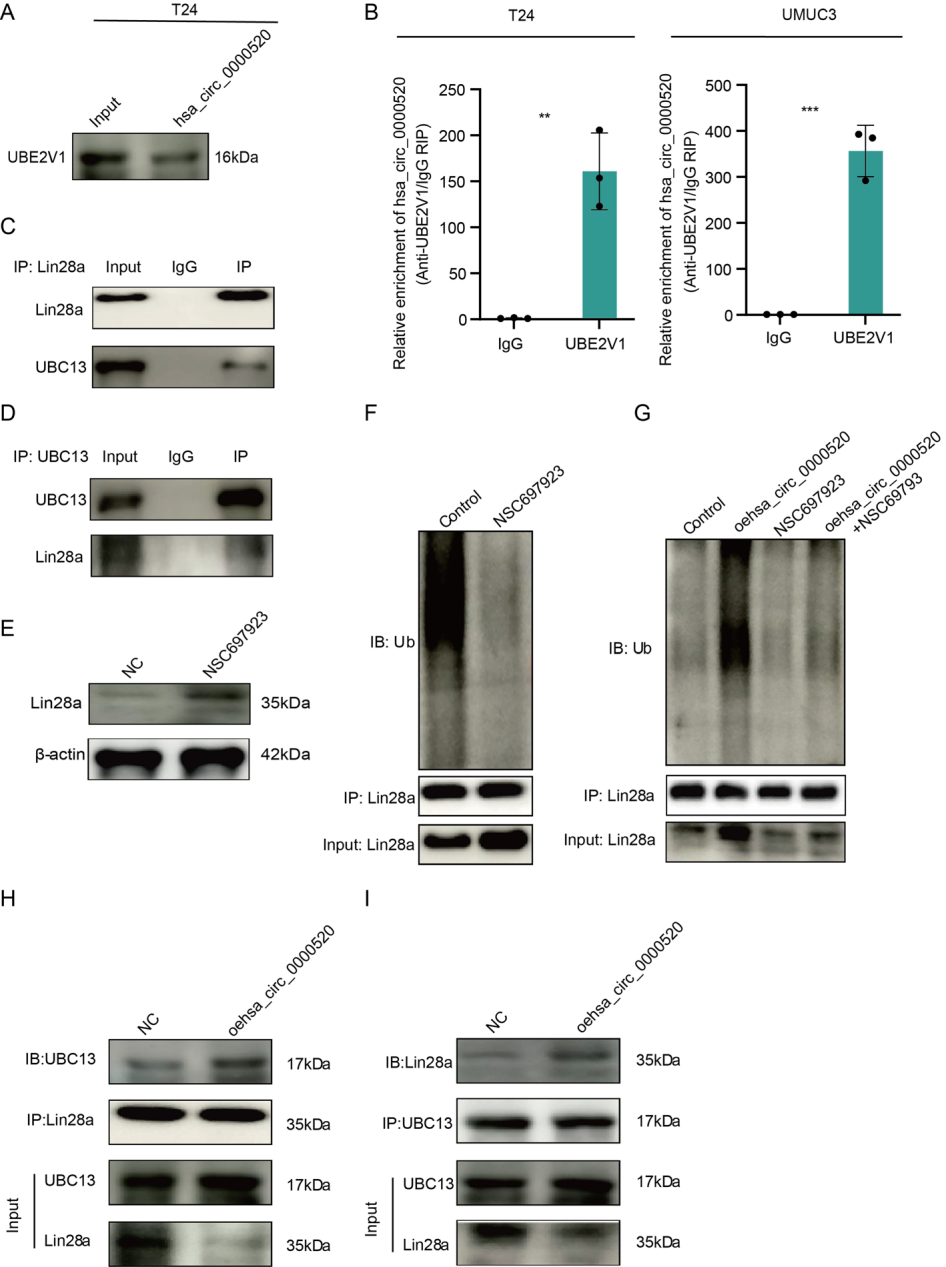
Hsa_circ_0000520 acts as a scaffold to promote the degradation of Lin28a by UBE2V1/UBC13. **A** RNA pulldown product validation by WB confirms the pull-down of UBE2V1 by hsa_circ_0000520. **B** RIP reverse analysis demonstrates the binding between UBE2V1 and hsa_circ_0000520. **C** co-IP analysis confirms the binding of UBC13 to Lin28a. **D** co-IP analysis validates the binding of Lin28a to UBC13. **E** WB analysis of changes in Lin28a protein levels after treatment with the UBE2V1-UBC13 inhibitor (NSC697923). **F** Co-IP analysis of changes in Lin28a ubiquitination levels after treatment with the UBE2V1-UBC13 inhibitor (NSC697923). **G** Co-IP analysis of the regulatory effect of the UBE2V1-UBC13 inhibitor (NSC697923) on Lin28a ubiquitination levels after overexpression of hsa_circ_0000520. **H**, **I** Co-IP analysis of changes in the binding ability between UBC13 and Lin28a after overexpression of hsa_circ_0000520. β-actin serves as an internal reference. *P < 0.05; **P < 0.01; ***P < 0.001; ****P < 0.0001

To further identify the regulatory mechanisms related to Lin28a ubiquitination, we reanalyzed the mass spectrometry results from the hsa_circ_0000520 pulldown. UBE2V1 was found among the potential interacting proteins. The interaction between hsa_circ_0000520 and UBE2V1 was further confirmed through Western blotting (WB) and RNA immunoprecipitation (RIP) experiments (Fig. 4A, B). UBE2V1 is one of the ubiquitin-conjugating enzyme E2 variant proteins, belonging to a unique subfamily of the E2 protein family with a structural feature lacking the conserved cysteine catalytic activity of E2 proteins. Previous studies have shown that the ubiquitination function of UBE2V1 can be mediated by UBC13 [18, 19]. We further validated the interaction between UBE2V1 and UBC13 in bladder cancer cells (Supplementary Fig. 2). Co-immunoprecipitation (co-IP) experiments in T24 cells revealed the interaction between UBC13 and Lin28a (Fig. 4C, D). Treatment of T24 cells with NSC697923, a small-molecule inhibitor of UBC13/UBE2V1, effectively attenuated the ubiquitination level of Lin28a, leading to an increase in Lin28a expression (Fig. 4E, F). These results suggest that UBE2V1/UBC13 interacts with Lin28a protein in bladder cancer cells, leading to degradation of Lin28a protein through the ubiquitin–proteasome pathway. Previous studies have indicated that some ubiquitin-related enzymes efficiently utilize RNA as a scaffold for ubiquitination [20, 21]. Therefore, we explored whether the ubiquitin ligase activity of UBE2V1/UBC13 depends on the presence of hsa_circ_0000520. Overexpression of hsa_circ_0000520 significantly attenuated the inhibitory effect of UBE2V1/UBC13 inhibitor on Lin28a ubiquitination degradation (Fig. 4G). To further validate these results and examine whether hsa_circ_0000520 acts as a scaffold to enhance the binding between UBC13 and Lin28a, co-IP analysis was performed (Fig. 4H, I), revealing that overexpression of hsa_circ_0000520 enhanced the binding between UBC13 and Lin28a. In summary, our research results indicate that hsa_circ_0000520 acts as a scaffold to enhance the interaction between UBE2V1/UBC13 and Lin28a, thereby promoting UBE2V1/UBC13-mediated ubiquitination degradation of Lin28a.

### Hsa_circ_0000520/Lin28a regulates the PI3K/AKT pathway by enhancing PTEN mRNA stability, thereby inhibiting bladder cancer invasion, migration, and VM formation

**Fig. 5:**
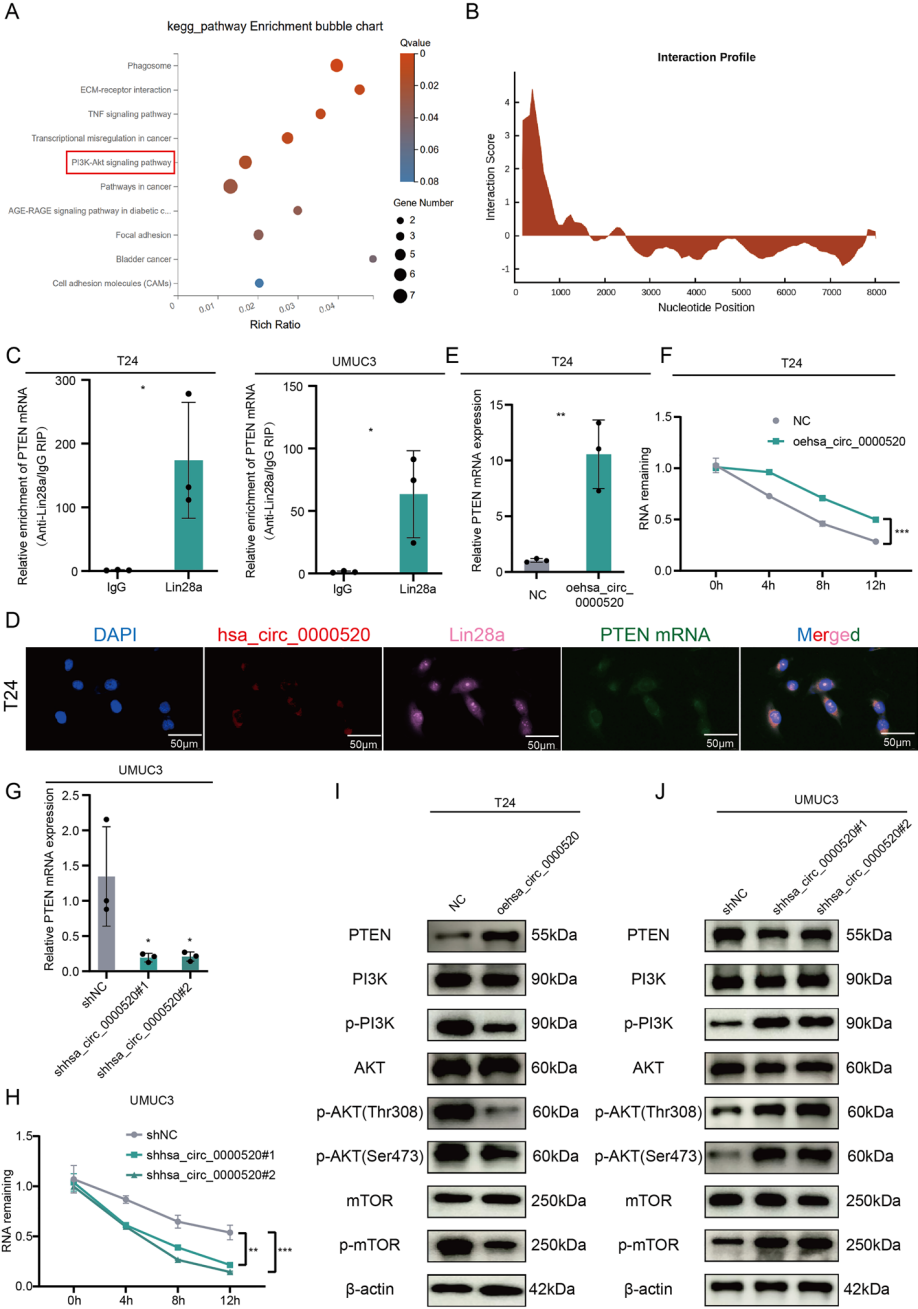
Hsa_circ_0000520 regulates the PI3K/AKT pathway by enhancing PTEN mRNA stability. **A** KEGG analysis of differentially expressed mRNAs in T24 cells with hsa_circ_0000520 knockdown compared with control. **B** catPARID prediction of the potential binding ability between Lin28a and PTEN mRNA. **C** RIP experiment validating the binding between Lin28a and PTEN mRNA. **D** Fluorescence co-localization experiment analyzing the relationship between hsa_circ_0000520, Lin28a, and PTEN mRNA. **E** qRT-PCR analysis of the impact of hsa_circ_0000520 overexpression on PTEN mRNA levels. **F** qRT-PCR analysis of changes in PTEN RNA abundance after treatment with actinomycin d in cells overexpressing hsa_circ_0000520. **G** qRT-PCR analysis of the impact of hsa_circ_0000520 knockdown on PTEN mRNA levels. **H** qRT-PCR analysis of changes in PTEN RNA abundance after treatment with actinomycin d in cells with hsa_circ_0000520 knockdown. **I** WB analysis of the impact of hsa_circ_0000520 overexpression on PTEN, PI3K, AKT, mTOR, p-PI3K, p-AKT, and p-mTOR protein levels. **J** WB analysis of the impact of hsa_circ_0000520 knockdown on PTEN, PI3K, AKT, mTOR, p-PI3K, p-AKT, and p-mTOR protein levels. β-actin serves as an internal reference. *P < 0.05; **P < 0.01; ***P < 0.001; ****P < 0.0001

**Fig. 6:**
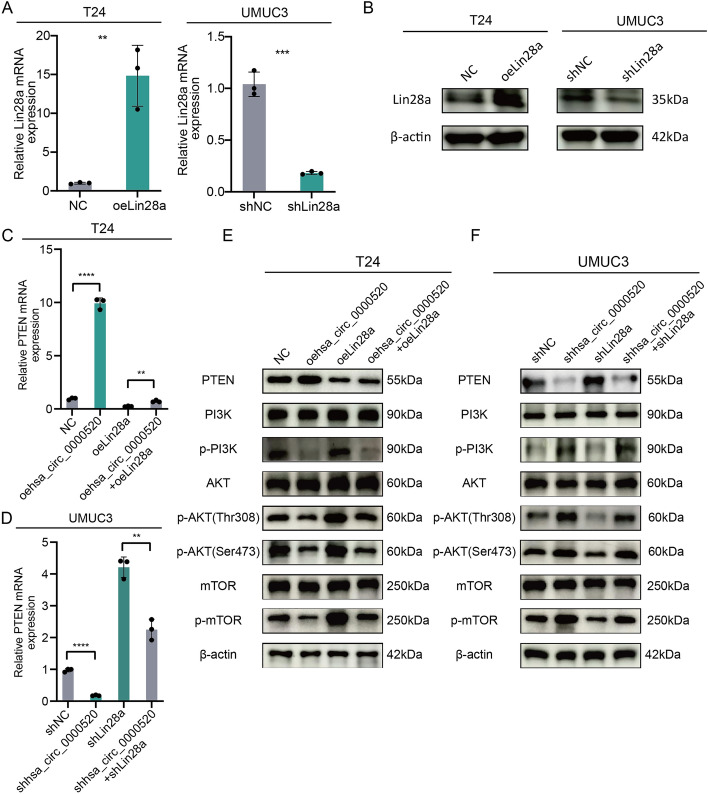
Hsa_circ_0000520/Lin28a regulates the PI3K/AKT pathway by enhancing PTEN mRNA stability. **A** qRT-PCR analysis validating the transfection efficiency of Lin28a overexpression and knockdown. **B** WB analysis confirming the transfection efficiency of Lin28a overexpression and knockdown. **C** qRT-PCR analysis showing that Lin28a overexpression partially reverses the impact of hsa_circ_0000520 overexpression on PTEN mRNA expression levels. **D** qRT-PCR analysis demonstrating that Lin28a knockdown partially reverses the impact of hsa_circ_0000520 knockdown on PTEN mRNA expression levels. **E** WB analysis revealing that Lin28a overexpression partially reverses the impact of hsa_circ_0000520 overexpression on PTEN, PI3K, AKT, mTOR, p-PI3K, p-AKT, and p-mTOR protein levels. **F** WB analysis indicating that Lin28a knockdown partially reverses the impact of hsa_circ_0000520 knockdown on PTEN, PI3K, AKT, mTOR, p-PI3K, p-AKT, and p-mTOR protein levels. β-actin serves as an internal reference. *P < 0.05; **P < 0.01; ***P < 0.001; ****P < 0.0001

To elucidate the functional role of hsa_circ_0000520 in bladder cancer, stable overexpression and knockdown cell lines of hsa_circ_0000520 were established in T24 and UMUC3 cells. Transcriptome sequencing and KEGG pathway enrichment analysis revealed significant enrichment in the PI3K-AKT pathway (Fig. 5A). Lin28a is an RNA-binding protein that can exert its effects by influencing target mRNAs involved in tumor progression [22, 23]. Lin28a’s potential binding to PTEN mRNA was predicted using catPARID (Fig. 5B). This prediction was validated through RIP and fluorescence co-localization experiments (Fig. 5C, D). Thus, we hypothesized that hsa_circ_0000520 affects PTEN expression and tumorigenesis by degrading Lin28a. Results showed that knockdown of hsa_circ_0000520 significantly decreased PTEN mRNA expression and stability, while overexpression of hsa_circ_0000520 increased PTEN mRNA expression and stability (Fig. 5E–H). Western blot analysis demonstrated that hsa_circ_0000520 affected key molecules in the PI3K-AKT pathway, such as PTEN, PI3K, AKT, mTOR, p-PI3K, p-AKT, and p-mTOR (Fig. 5I, J). Overexpression of hsa_circ_0000520 increased PTEN protein levels, with no significant change in the protein levels of PI3K, AKT, and mTOR, but a decrease in their phosphorylation levels. Knockdown of hsa_circ_0000520 significantly inhibited PTEN protein levels and increased the phosphorylation levels of PI3K, AKT, and mTOR in bladder cancer cells. Subsequently, overexpression and knockdown of Lin28a were performed in T24 and UMUC3 cells. The efficiency is shown in Fig. 6A, B. Further qRT-PCR and Western blot analyses indicated that overexpression of Lin28a inhibited the increase in PTEN mRNA and protein levels caused by hsa_circ_0000520, while knockdown of Lin28a reversed the impact of hsa_circ_0000520 knockdown on PTEN mRNA and protein levels (Fig. 6C, D). Additionally, Lin28a reversed the effects of hsa_circ_0000520 on the phosphorylation levels of PI3K, AKT, and mTOR (Fig. 6E, F). These results collectively suggest that the interaction between hsa_circ_0000520 and Lin28a promotes PTEN expression, thereby inhibiting the activation of the PI3K/AKT/mTOR pathway. Subsequent rescue experiments were performed in T24 and UMUC3 cells. Transwell and scratch assays showed that overexpression of Lin28a partially reversed the inhibitory effect of hsa_circ_0000520 on cell migration and invasion, while knockdown of Lin28a partially inhibited the enhanced invasiveness of bladder cancer caused by hsa_circ_0000520 knockdown (Supplementary Fig. 3A–D). Moreover, VM formation assays demonstrated that Lin28a could rescue the impact of hsa_circ_0000520 on bladder cancer VM formation (Supplementary Fig. 3E, F). In summary, these findings indicate that hsa_circ_0000520 inhibits bladder cancer invasion, migration, and VM formation by suppressing the Lin28a/PTEN/PI3K/AKT axis.

### In vivo validation of hsa_circ_0000520/Lin28a suppression of bladder cancer metastasis

**Fig. 7:**
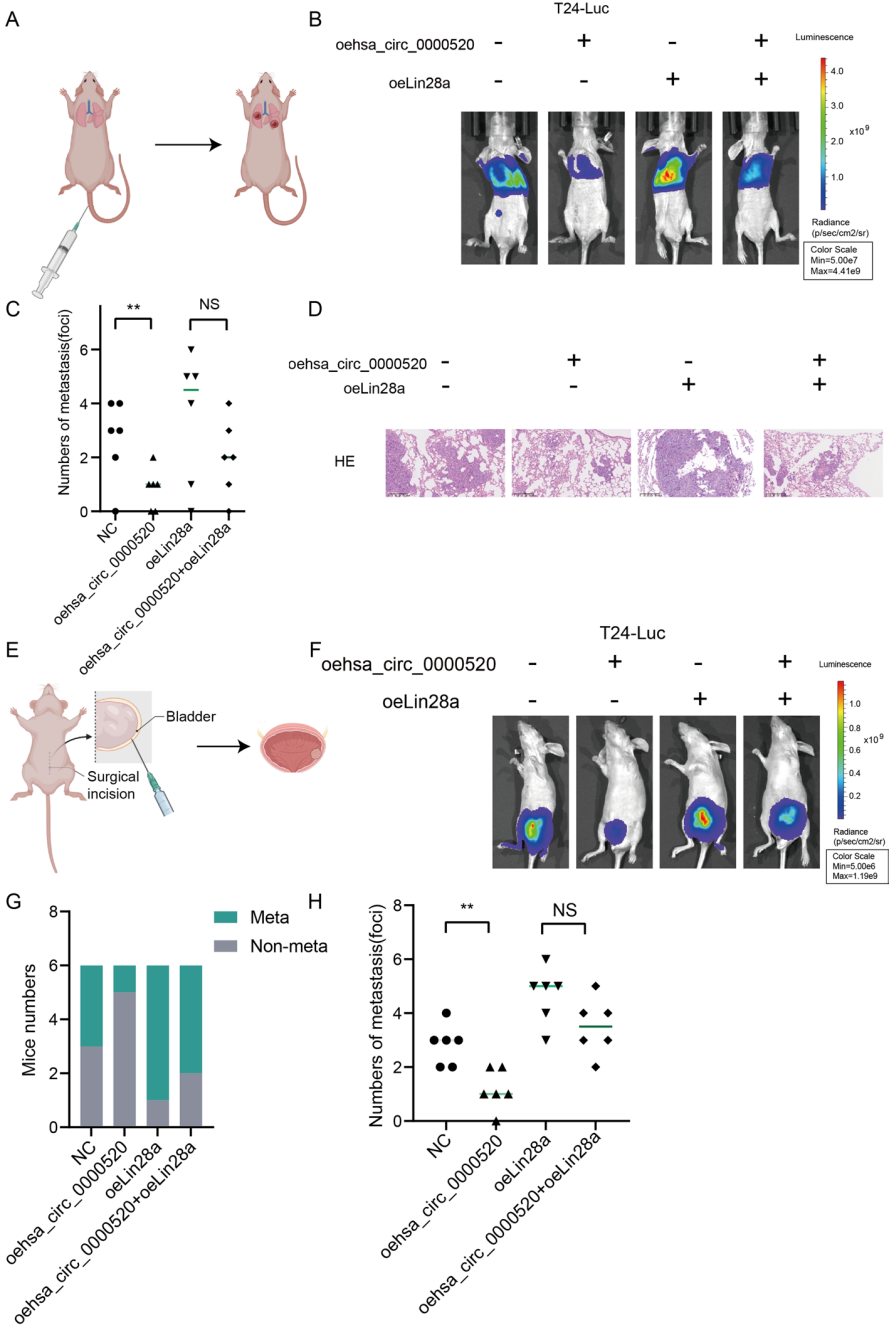
In vivo experiments validate the inhibitory effect of hsa_circ_0000520/ Lin28a on bladder cancer metastasis. **A** Schematic representation of the construction of the nude mouse lung metastasis model (Image elements from Biorender). **B** In vivo bioluminescence imaging analysis of lung metastasis in mice from each group. **C** Statistical analysis of the differences in the number of lung metastatic tumors in each group of mice. **D** Lung tissue sections stained with hematoxylin and eosin (HE) to analyze the tumor status in mice from each group. **E** Schematic representation of the construction of the nude mouse orthotopic bladder cancer transplantation model (Image elements from Biorender). **F** In vivo bioluminescence imaging analysis of tumor growth and metastasis in mice from each group. **G** Statistical analysis of the number of mice with or without metastasis in each group. **H** Statistical analysis of the differences in the number of tumor metastases in each group of mice. *P < 0.05; **P < 0.01; ***P < 0.001; ****P < 0.0001

To better illustrate the role of hsa_circ_0000520 in bladder cancer metastasis, we employed T24 cells overexpressing hsa_circ_0000520, Lin28a, or both hsa_circ_0000520 and Lin28a (luciferase-labeled). These cells were then injected into the tail vein of nude mice to allow seeding in the lungs, thereby establishing a lung metastasis model. In vivo bioluminescence imaging revealed a significant reduction in luciferase activity in the hsa_circ_0000520 overexpression group. Bladder tumor weights collected showed a decrease in the hsa_circ_0000520 overexpression group, and H&E staining showed a noticeable decrease in tumor formation. PTEN mRNA and protein levels both increased. Additionally, mice in the hsa_circ_0000520 overexpression group exhibited extended survival times. However, this effect was partially reversed by Lin28a overexpression (Fig. 7A–D, Supplementary Fig. 4). These data indicate that hsa_circ_0000520 inhibits cancer metastasis in vivo, consistent with the in vitro results. Furthermore, luciferase-labeled T24 cells overexpressing hsa_circ_0000520, Lin28a, or both were orthotopically implanted into the bladder wall of nude mice to establish orthotopic transplantation bladder cancer. After 6 weeks of implantation, the mice were euthanized, and primary tumors and metastatic foci were examined. Results showed a significant reduction in metastatic tumors in the hsa_circ_0000520 overexpression group. Additionally, in vivo imaging results were consistent with the aforementioned lung metastasis model results (Fig. 7E–H). In conclusion, our study results suggest that hsa_circ_0000520 inhibits bladder cancer metastasis through Lin28a.

### QKI promotes the formation of hsa_circ_0000520

**Fig. 8:**
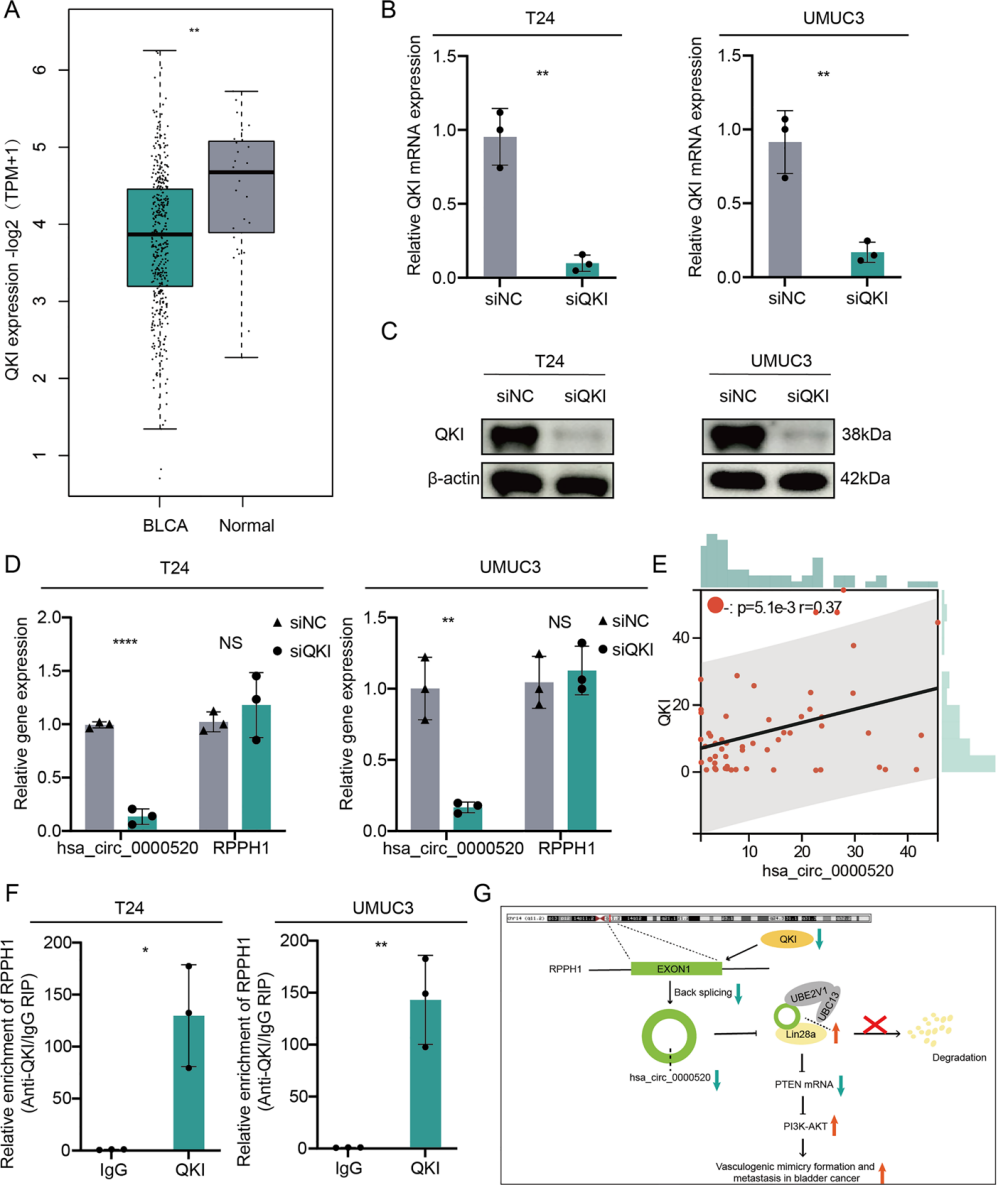
QKI promotes the formation of hsa_circ_0000520. **A** Analysis of QKI expression differences between bladder cancer and normal tissues in the TCGA dataset. **B** qRT-PCR to detect the transfection efficiency of QKI knockdown. **C** WB analysis of the transfection efficiency of QKI knockdown. **D** qRT-PCR analysis of the impact of QKI knockdown on hsa_circ_0000520 expression. **E** qRT-PCR analysis of the correlation between QKI and hsa_circ_0000520 expression in bladder cancer tissues. **F** RIP validation of the interaction between RPPH1 and QKI. **G** Schematic diagram of the mechanism in this study. β-actin serves as an internal control. *P < 0.05; **P < 0.01; ***P < 0.001; ****P < 0.0001

Hsa_circ_0000520 is derived from the splicing of RPPH1. We analyzed the expression and survival prognosis of RPPH1 in bladder cancer using TCGA public data and found no significant differences (Supplementary Fig. 5A–E). To further explore the upstream regulatory mechanism of hsa_circ_0000520, we first predicted proteins that interact with RPPH1 through RBPDP and catPARID. We then intersected these predictions with seven well-established RNA-binding proteins (ADAR, ADAR2, DHX9, QKI, MBL, FUS, and HNRNPL) involved in circRNA formation, obtaining FUS and QKI as potential regulators in the alternative splicing process of hsa_circ_0000520 (Supplementary Fig. 5F). Evaluation of the expression of these two genes in the TCGA bladder cancer dataset revealed a significant downregulation of QKI in bladder cancer, consistent with the expression trend of hsa_circ_0000520. However, FUS showed no significant difference in expression between bladder cancer and adjacent tissues (Supplementary Fig. 5G, Fig. 8A). Therefore, we hypothesized that QKI plays a crucial role in the formation of hsa_circ_0000520. T24 or UMUC3 cells were transfected with QKI siRNA, and qRT-PCR and Western blot were performed to assess the transfection efficiency (Fig. 8B, C). The results indicated that knockdown of QKI significantly decreased the expression of hsa_circ_0000520 (Fig. 8D). Furthermore, qRT-PCR analysis of 56 bladder cancer tissues showed a positive correlation between QKI and hsa_circ_0000520 at the mRNA expression level (Fig. 8E). RIP experiments confirmed the interaction between RPPH1 and QKI (Fig. 8F). Additionally, potential binding sites and sequences were predicted using RBPsuit and catPARID (Supplementary Fig. 6). Thus, our findings suggest that QKI promotes the splicing of RPPH1, leading to the formation of hsa_circ_0000520.

## Discussion

High-throughput sequencing has been employed to identify circRNAs, including those acting as tumor suppressors or oncogenes in human cancers [24]. However, the unique role of circRNAs in the occurrence and development of bladder cancer is not fully understood and requires further investigation. In this study, we observed that hsa_circ_0000520, derived from the lncRNA RPPH1, was downregulated in bladder cancer cell lines and tissues. Overexpression of hsa_circ_0000520 inhibited the invasion, migration, and vasculogenic mimicry formation of bladder cancer cells. High expression of hsa_circ_0000520 predicted better prognosis for bladder cancer patients.

Previous studies have indicated that circRNAs play various crucial roles in cellular physiology by acting as miRNA sponges, binding molecules for RBPs, transcriptional regulators, or protein translation templates [25]. In this study, we discovered through RNA pulldown and RIP experiments that hsa_circ_0000520 interacts with Lin28a, suggesting a potential interaction between them. Moreover, hsa_circ_0000520 primarily influences the protein level of Lin28a rather than its transcriptional level. Some ubiquitin-related enzymes efficiently utilize RNA as a scaffold for ubiquitination [21]. Li et al. found that circNDUFB2 acts as a scaffold to enhance the interaction between TRIM25 and IGF2BPs, promoting the ubiquitination degradation of IGF2BPs by TRIM25 [26]. Analysis of the hsa_circ_0000520 pulldown mass spectrometry results revealed an interaction between UBE2V1 and hsa_circ_0000520. UBE2V1 is one of the ubiquitin-conjugating enzyme E2 variant proteins, belonging to a unique subfamily within the E2 protein family, lacking the conserved cysteine catalytic activity found in typical E2 proteins. Studies have suggested that the ubiquitination function of UBE2V1 is mediated by UBC13 [18, 19]. After treating bladder cancer cells with a UBE2V1/UBC13 inhibitor, both ubiquitination and expression of Lin28a were affected, and UBC13 was found to interact with Lin28a, indicating that UBE2V1/UBC13 regulates the ubiquitination of Lin28a. Additionally, the overexpression of hsa_circ_0000520 weakened the inhibitory effect of the UBE2V1/UBC13 inhibitor on the ubiquitination level of Lin28a. The overexpression of hsa_circ_0000520 enhanced the interaction between UBC13 and Lin28a. These results suggest that hsa_circ_0000520 may act as a scaffold, enhancing the interaction between UBE2V1/UBC13 and Lin28a, promoting the ubiquitination degradation of Lin28a by UBE2V1/UBC13.

Alternative splicing of pre-mRNA is a crucial biological feature in eukaryotes [27]. QKI has been shown to regulate pre-mRNA splicing [28]. It is noteworthy that the insertion of QKI binding motifs can induce the formation of circRNA. The close proximity of exons in pre-mRNA secondary structures promotes the biogenesis of circRNA [29]. A previous study indicated that QKI targets exonic flanking introns of BCAR3 [30], SMARCA5 [31], and NDUFB2 [26] to facilitate circRNA formation. Based on these characteristics, we predicted potential binding sites for RPPH1 and QKI using catPARID and RBPsuit. We identified a QKI binding sequence near hsa_circ_0000520, suggesting that QKI promotes the formation of hsa_circ_0000520. RIP experiments confirmed the binding of RPPH1 to QKI, and the expression of hsa_circ_0000520 decreased upon QKI knockdown.

Angiogenesis, a process mimicking blood vessel formation, is one of the key factors contributing to malignant progression and drug resistance in tumors. However, the underlying mechanisms and targeted drug research in this regard remain unclear. In this study, we aimed to investigate the crucial role of circRNA in bladder cancer angiogenesis and metastasis through in vitro and in vivo experiments, as well as through techniques such as RNA immunoprecipitation (RIP), RNA pulldown, co-immunoprecipitation (co-IP), and immunofluorescence co-localization. CircRNAs possess stable, closed-loop, and conserved characteristics. With increasing understanding of circRNA and advancements in biosynthesis techniques, recent studies have successfully synthesized circRNA in vitro, opening up potential clinical applications [32]. In the future, further in vitro synthesis of hsa_circ_0000520 and its application in animal models will be conducted to explore its potential therapeutic effects.

However, our study has some limitations. Currently, we have only evaluated the expression of hsa_circ_0000520 in 30 pairs of bladder cancer and adjacent tissues. To fully confirm the inhibitory role of hsa_circ_0000520, future research needs to expand to more bladder cancer samples and adjacent tissue samples. Additionally, the specific binding sites between hsa_circ_0000520 and Lin28a, as well as the functional sites of QKI with hsa_circ_0000520, require further investigation. Future efforts will focus on synthesizing hsa_circ_0000520 in vitro and validating its potential therapeutic effects in animal models. This is the direction where our research needs further exploration.

## Conclusions

In this study, we identified a circRNA derived from the *RPPH1* gene, namely hsa_circ_0000520. Hsa_circ_0000520 was associated with better prognosis in bladder cancer patients. Functionally, hsa_circ_0000520 inhibited bladder cancer invasion, migration, and vasculogenic mimicry (VM) formation. In summary, our study delineated the significant role of hsa_circ_0000520 in tumor progression and vasculogenic mimicry, providing a potential therapeutic target to overcome bladder cancer metastasis and resistance to antiangiogenic drugs.

## Supplementary Information

Supplementary Material 1. Figure 1. Clinical specimen analysis reveals a significant negative correlation between hsa_circ_0000520 and VM formation.Bladder cancer tissue microarray with dual staining for CD31 immunohistochemistry and PAS staining, demonstrating endothelial vessels and vasculogenic mimicrystructures.Fluorescence in situ hybridization, CD31 immunohistochemistry, and PAS staining dual staining on bladder cancer tissue microarray, illustrating the number of VM formations in bladder cancer with varying expression levels of hsa_circ_0000520.Correlation analysis between the expression level of hsa_circ_0000520 and the number of VM formations. *P < 0.05; **P < 0.01; ***P < 0.001; ****P < 0.0001.

Supplementary Material 2. Figure 2. Verification of the interaction between UBE2V1 and UBC13.co-IP analysis confirms the binding of UBE2V1 to UBC13.co-IP analysis validates the binding of UBC13 to UBE2V1

Supplementary Material 3. Figure 3. In vitro validation of hsa_circ_0000520 inhibiting bladder cancer invasion, migration, and VM formation through Lin28a.Scratch assay showing that overexpression of Lin28a partially reverses the inhibitory effect of hsa_circ_0000520 overexpression on bladder cancer cell migration.Transwell assay demonstrating that overexpression of Lin28a partially reverses the inhibitory effects of hsa_circ_0000520 overexpression on bladder cancer cell migration, invasion.VM formation assay indicating that overexpression of Lin28a partially reverses the inhibitory effect of hsa_circ_0000520 overexpression on bladder cancer cell VM formation.Scratch assay revealing that knockdown of Lin28a partially reverses the inhibitory effect of hsa_circ_0000520 knockdown on bladder cancer cell migration.Transwell migration assay showing that knockdown of Lin28a partially reverses the inhibitory effects of hsa_circ_0000520 knockdown on bladder cancer cell migration and invasion.VM formation assay demonstrating that knockdown of Lin28a partially reverses the inhibitory effect of hsa_circ_0000520 knockdown on bladder cancer cell VM formation. *P < 0.05; **P < 0.01; ***P < 0.001; ****P < 0.0001.

Supplementary Material 4. Figure 4. In vivo validation of the effects of hsa_circ_0000520 on PTEN expression, tumor growth, and survival in mice.qRT-PCR analysis of PTEN mRNA levels in bladder tumors from each group of mice.WB analysis of PTEN levels in bladder tumors from each group of mice.The bladder tumor weight in each group of mice.The survival status of mice in each group

Supplementary Material 5. Figure 5. Expression and prognosis analysis of RPPH1 in bladder cancer.Analysis of RPPH1 expression differences between bladder cancer and normal tissues in the TCGA dataset.Relationship between RPPH1 expression and the prognosis of bladder cancer patients in the TCGA dataset.Venn diagram showing the intersection of predicted results from RBPDP and catPARID.Analysis of FUS expression differences between bladder cancer and normal tissues in the TCGA dataset. *P < 0.05; **P < 0.01; ***P < 0.001; ****P < 0.0001.

Supplementary Material 6. Figure 6. Prediction of QKI binding to RPPH1.Prediction of QKI binding sites on RPPH1 using RBPsuite.Prediction of QKI binding sequences on RPPH1 using catPARID

Supplementary Material 7. Table 1. Primer sequence list. Table 2. Correlation analysis between the expression of hsa_circ_0000520 and clinicopathological features in bladder cancer. Table 3. The mass spectrometry result of hsa_circ_0000520 pulldown

## Data Availability

Data are available from the corresponding author upon reasonable request.

## Abbreviations

VM: Vasculogenic mimicry
BCa: Bladder cancer
NMIBC: Non-muscle-invasive bladder cancer
MIBC: Muscle invasive bladder cancer
circRNA: Circular RNA
gDNA: Genomic DNA
qRT-PCR: Quantitative real-time polymerase chain reaction
WB: Western blot
FISH: Fluorescence in situ hybridization
RIP: RNA binding protein immunoprecipitation
CHX: Cycloheximide
ACTD: Actinomycin d
IVIS: In vivo imaging system
OS: Overall survival
PAS: Periodic acid−Schiff
co-IP: Co-immunoprecipitation
