# Supplementary figures and images for: Hsa_circ_0000520 suppresses vasculogenic mimicry formation and metastasis in bladder cancer through Lin28a/PTEN/PI3K signaling
Source: Cell Mol Biol Lett. 2024 Sep 5;29:118. doi: 10.1186/s11658-024-00627-0 (PMC11378395; doi:10.1186/s11658-024-00627-0)

A

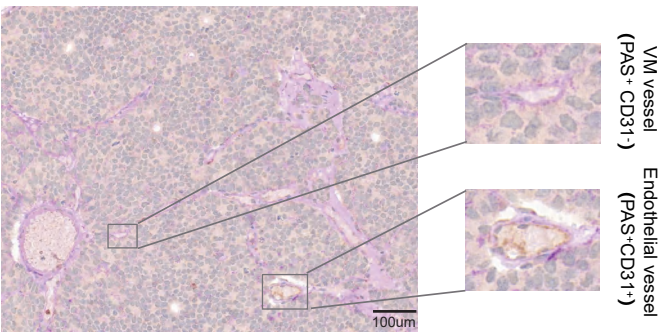

C

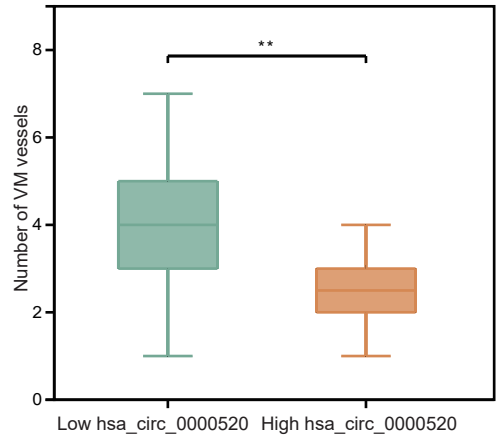

B

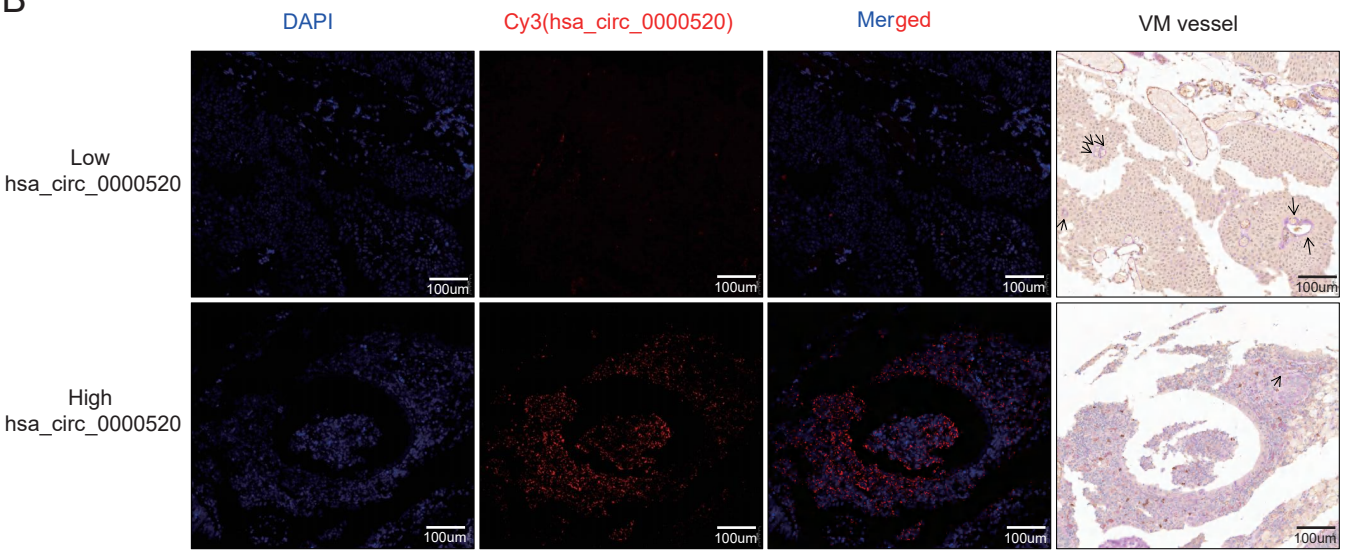

Supplement: Supplementary file 1 — Supplementary Material 1. Figure 1. Clinical specimen analysis reveals a significant negative correlation between hsa_circ_0000520 and VM formation.Bladder cancer tissue microarray with dual staining for CD31 immunohistochemistry and PAS staining, demonstrating endothelial vessels and vasculogenic mimicrystructures.Fluorescence in situ hybridization, CD31 immunohistochemistry, and PAS staining dual staining on bladder cancer tissue microarray, illustrating the number of VM formations in bladder cancer with varying expression levels of hsa_circ_0000520.Correlation analysis between the expression level of hsa_circ_0000520 and the number of VM formations. *P < 0.05; **P < 0.01; ***P < 0.001; ****P < 0.0001. [file 11658_2024_627_MOESM1_ESM.pdf]

A

IP: UBE2V1

Input

IgG

IP

UBE2V1

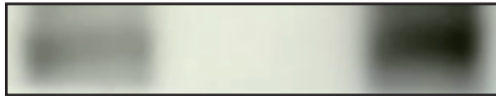

UBC13

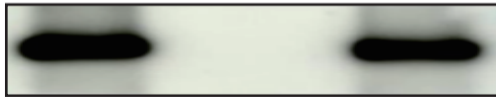

B

IP: UBC13

Input

IgG

IP

UBC13

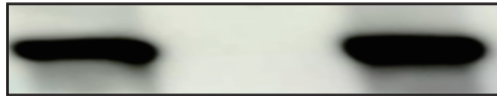

UBE2V1

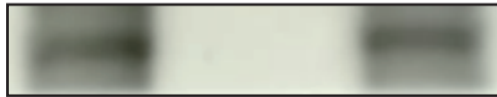

Supplement: Supplementary file 2 — Supplementary Material 2. Figure 2. Verification of the interaction between UBE2V1 and UBC13.co-IP analysis confirms the binding of UBE2V1 to UBC13.co-IP analysis validates the binding of UBC13 to UBE2V1 [file 11658_2024_627_MOESM2_ESM.pdf]

**A**

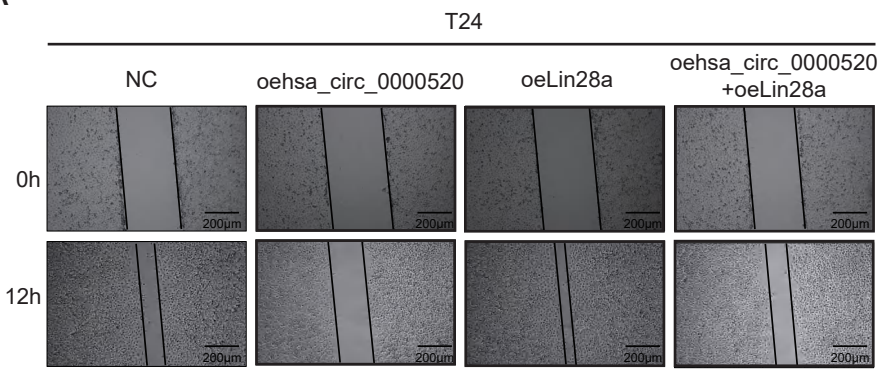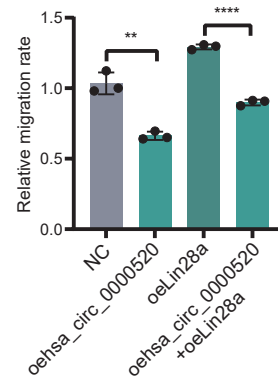

**B**

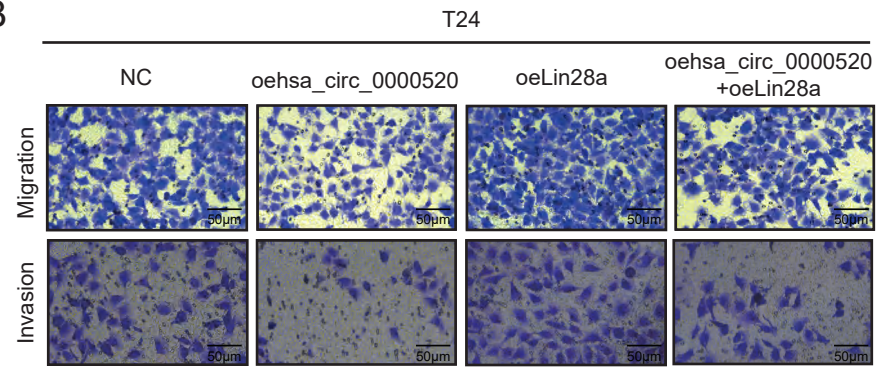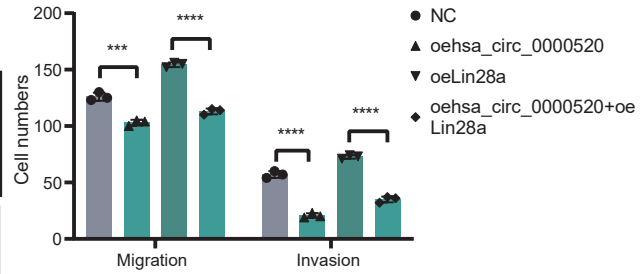

**C**

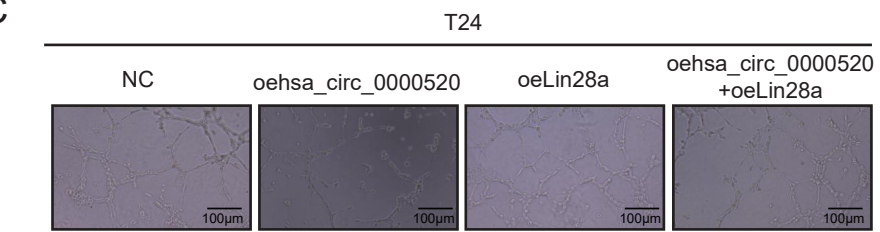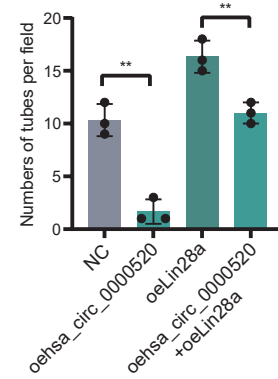

**D**

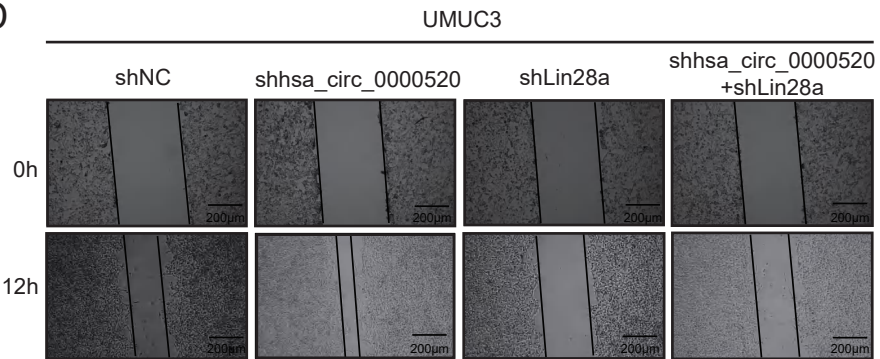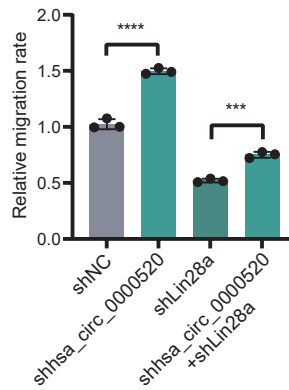

**E**

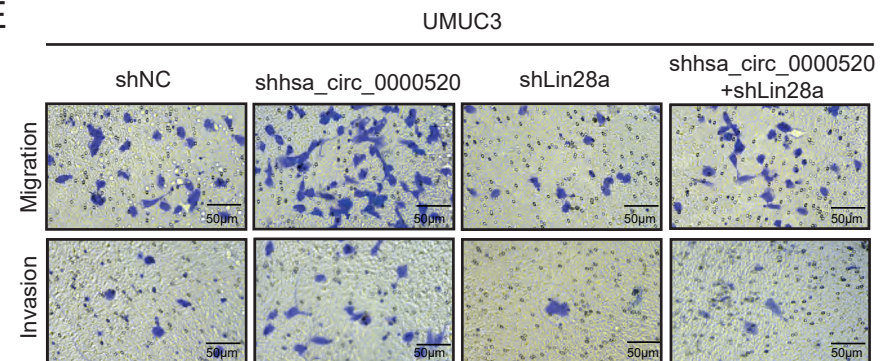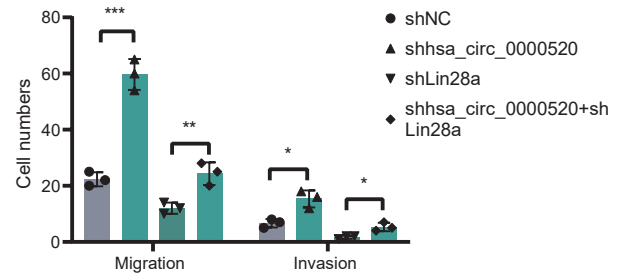

**F**

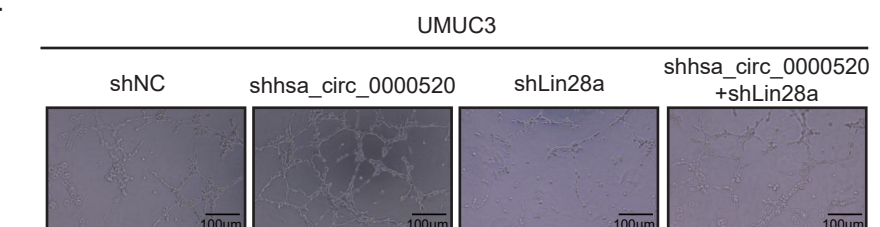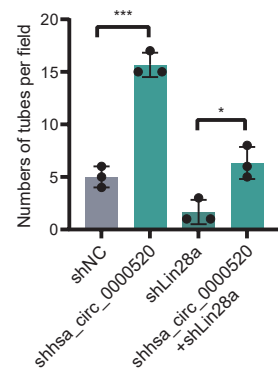

Supplement: Supplementary file 3 — Supplementary Material 3. Figure 3. In vitro validation of hsa_circ_0000520 inhibiting bladder cancer invasion, migration, and VM formation through Lin28a.Scratch assay showing that overexpression of Lin28a partially reverses the inhibitory effect of hsa_circ_0000520 overexpression on bladder cancer cell migration.Transwell assay demonstrating that overexpression of Lin28a partially reverses the inhibitory effects of hsa_circ_0000520 overexpression on bladder cancer cell migration, invasion.VM formation assay indicating that overexpression of Lin28a partially reverses the inhibitory effect of hsa_circ_0000520 overexpression on bladder cancer cell VM formation.Scratch assay revealing that knockdown of Lin28a partially reverses the inhibitory effect of hsa_circ_0000520 knockdown on bladder cancer cell migration.Transwell migration assay showing that knockdown of Lin28a partially reverses the inhibitory effects of hsa_circ_0000520 knockdown on bladder cancer cell migration and invasion.VM formation assay demonstrating that knockdown of Lin28a partially reverses the inhibitory effect of hsa_circ_0000520 knockdown on bladder cancer cell VM formation. *P < 0.05; **P < 0.01; ***P < 0.001; ****P < 0.0001. [file 11658_2024_627_MOESM3_ESM.pdf]

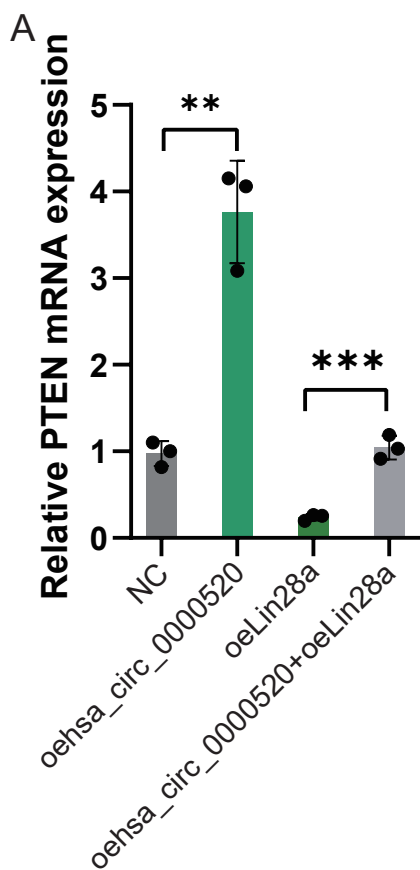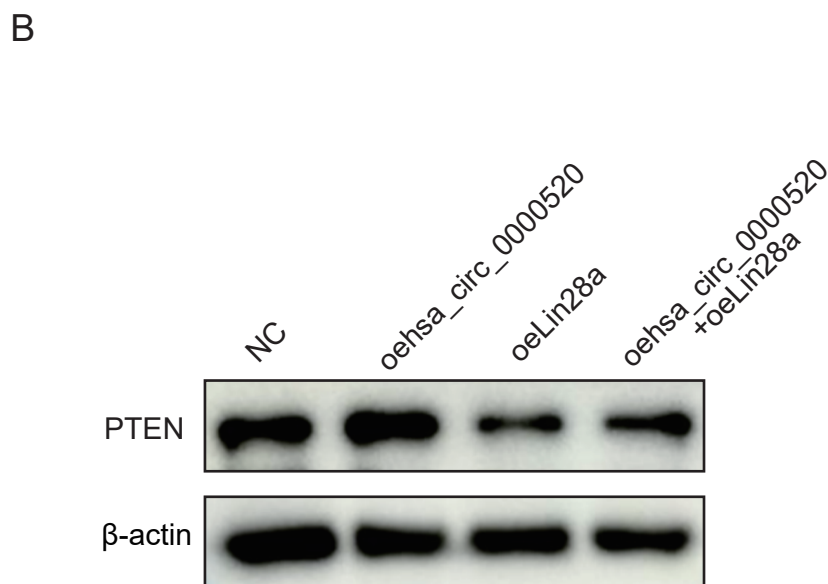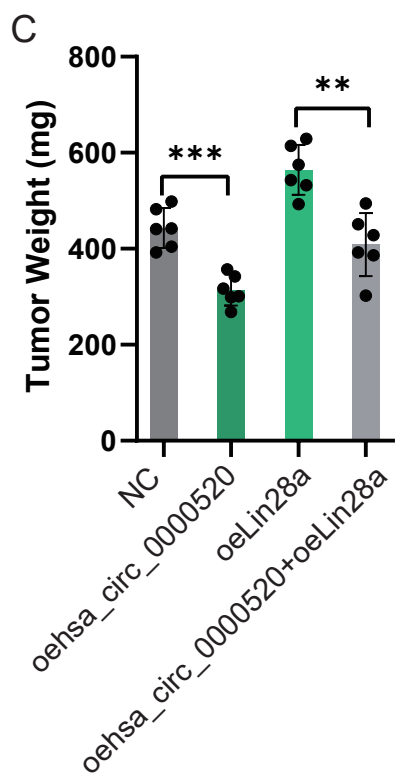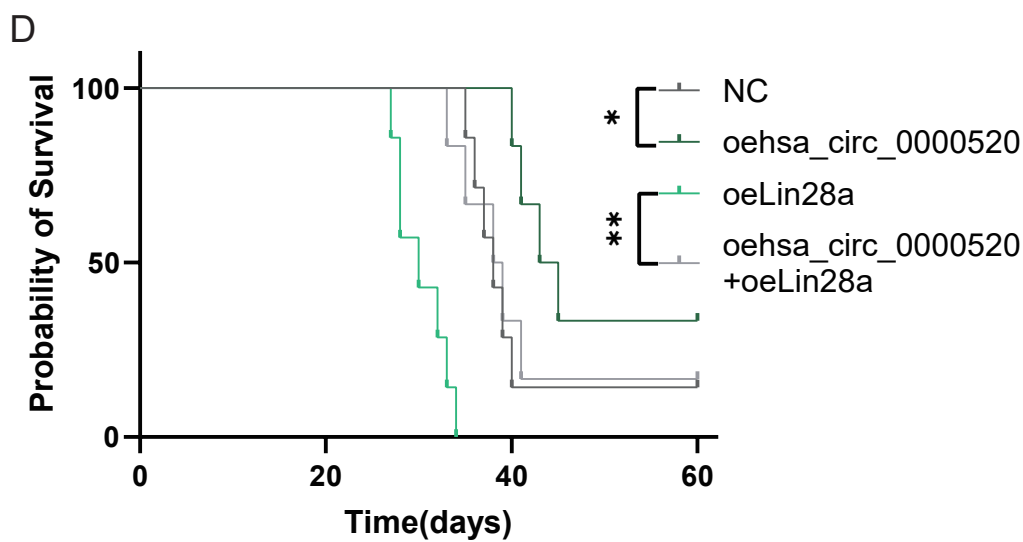

Supplement: Supplementary file 4 — Supplementary Material 4. Figure 4. In vivo validation of the effects of hsa_circ_0000520 on PTEN expression, tumor growth, and survival in mice.qRT-PCR analysis of PTEN mRNA levels in bladder tumors from each group of mice.WB analysis of PTEN levels in bladder tumors from each group of mice.The bladder tumor weight in each group of mice.The survival status of mice in each group [file 11658_2024_627_MOESM4_ESM.pdf]

A

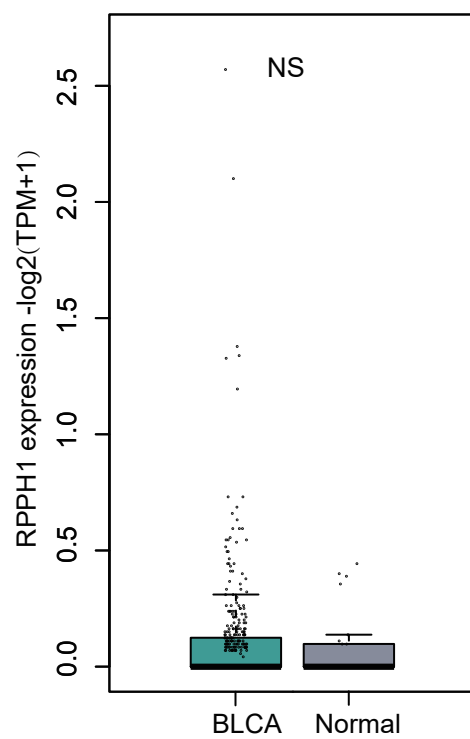

B

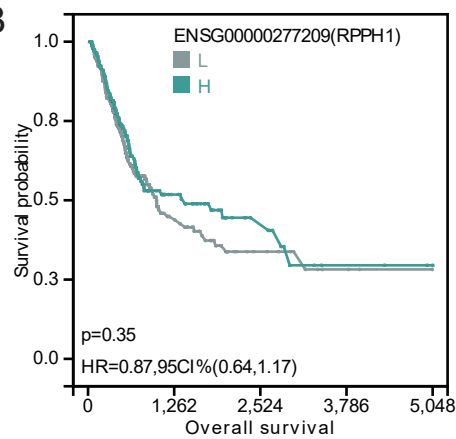

C

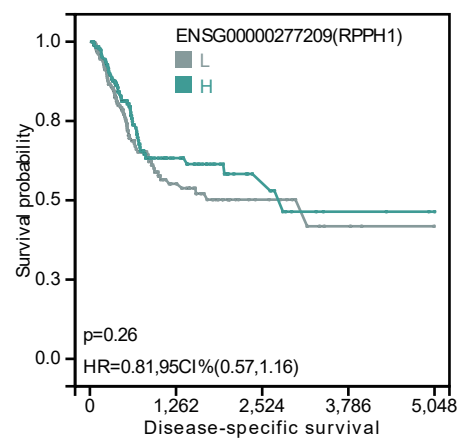

D

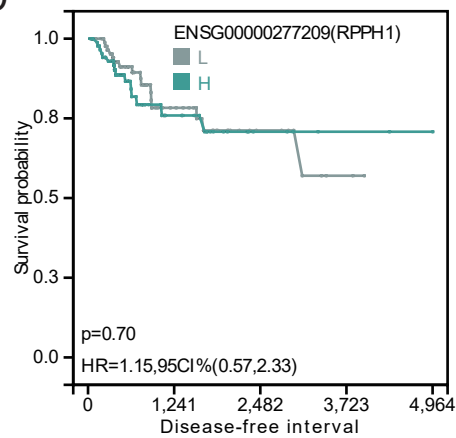

E

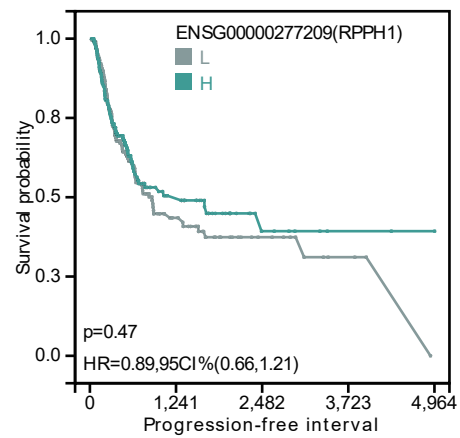

G

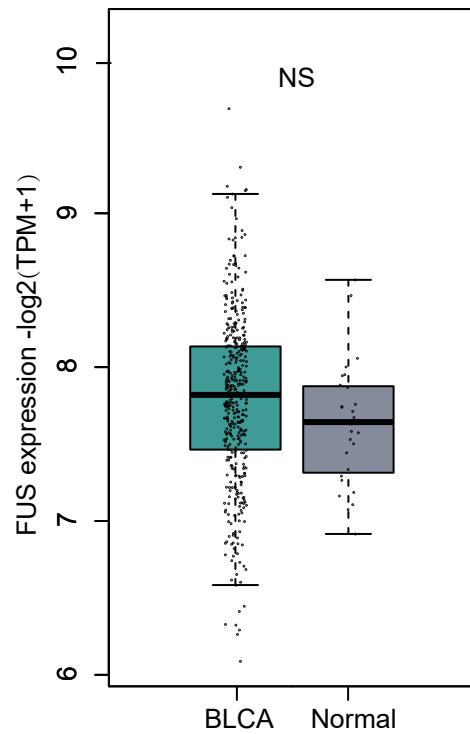

H

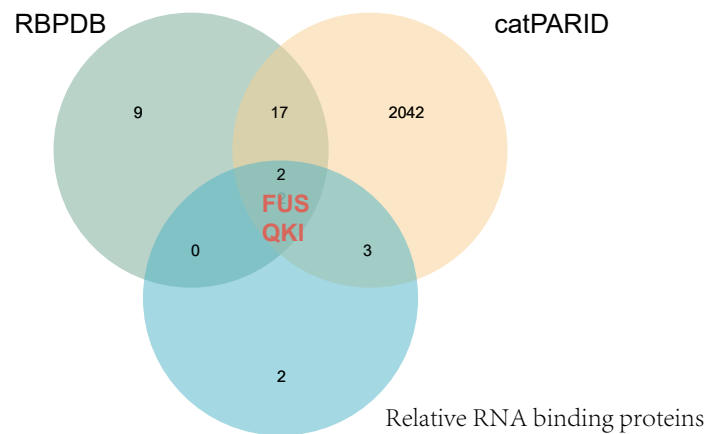

Supplement: Supplementary file 5 — Supplementary Material 5. Figure 5. Expression and prognosis analysis of RPPH1 in bladder cancer.Analysis of RPPH1 expression differences between bladder cancer and normal tissues in the TCGA dataset.Relationship between RPPH1 expression and the prognosis of bladder cancer patients in the TCGA dataset.Venn diagram showing the intersection of predicted results from RBPDP and catPARID.Analysis of FUS expression differences between bladder cancer and normal tissues in the TCGA dataset. *P < 0.05; **P < 0.01; ***P < 0.001; ****P < 0.0001. [file 11658_2024_627_MOESM5_ESM.pdf]

A

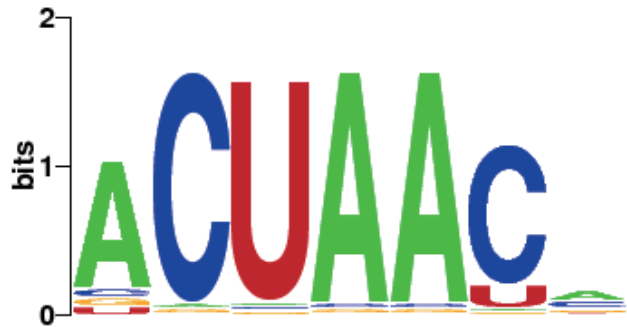

B

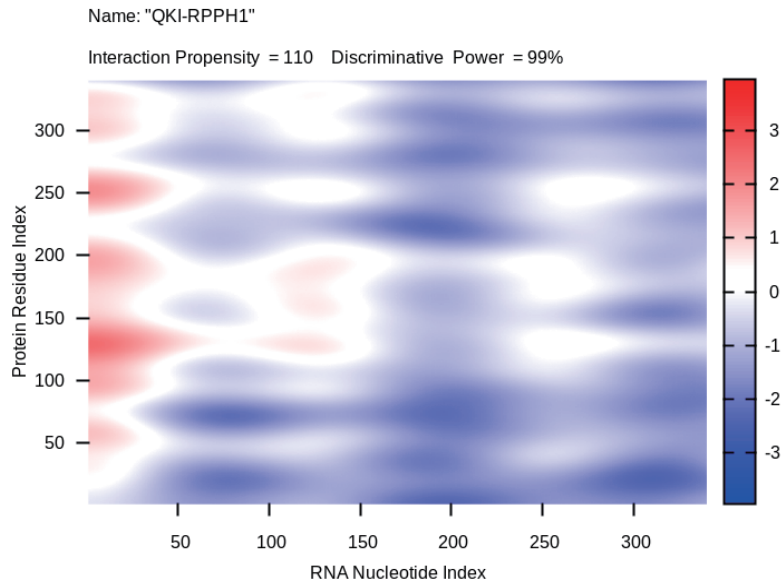

Supplement: Supplementary file 6 — Supplementary Material 6. Figure 6. Prediction of QKI binding to RPPH1.Prediction of QKI binding sites on RPPH1 using RBPsuite.Prediction of QKI binding sequences on RPPH1 using catPARID [file 11658_2024_627_MOESM6_ESM.pdf]
